# Up/down frunk: comparing recycling methods for non-woven poly(ethylene terephthalate) from an electric vehicle

**DOI:** 10.1039/d6lp00330c

**Published:** 2026-10-01

**Authors:** Claire L. Seitzinger, Lynn Anderson, Fabian Mehner, Helena Simmons, Ciaran W. Lahive, Michael P. Shaver

**Affiliations:** a Department of Materials, The University of Manchester Manchester UK michael.shaver@manchester.ac.uk; b Polestar Automotive UK Ltd Envisage Unit F Coventry UK

## Abstract

When assessing optimal recycling routes for a specific plastic product, the object geometry and formulation complexity play a significant role. For example, poly(ethylene terephthalate), PET, depolymerization processes that work well on bottles may not apply well to other sectors. Here, we compare the technical feasibility of two recycling methods for a non-woven PET textile from an electric vehicle. We consider thermomechanical recycling through shredding and re-extruding to produce a new filament, as well as chemical recycling by glycolysis. The success of each technique and the scale up thereof is compared, along with the need for pretreatment. Thermomechanical recycling proved challenging, as the material did not feed consistently into the extruder, resulting in an inconsistent and degraded filament. Glycolysis was successful, and indeed faster than when utilizing bottle feedstocks, and could be readily scaled to 1 kg of material. Therefore, for a non-woven PET with a carbon black colorant, glycolysis performed better than mechanical processing, highlighting the need to consider object-level technical feasibility alongside sustainability when proposing recycling methods.

## Abstract 2

An electric vehicle frunk, formed from non-woven PET, can have multiple imagined fates. This paper explores the opportunities and limitations of both mechanical recycling and chemical depolymerization of this real-world automotive waste stream.

## Graphical abstract

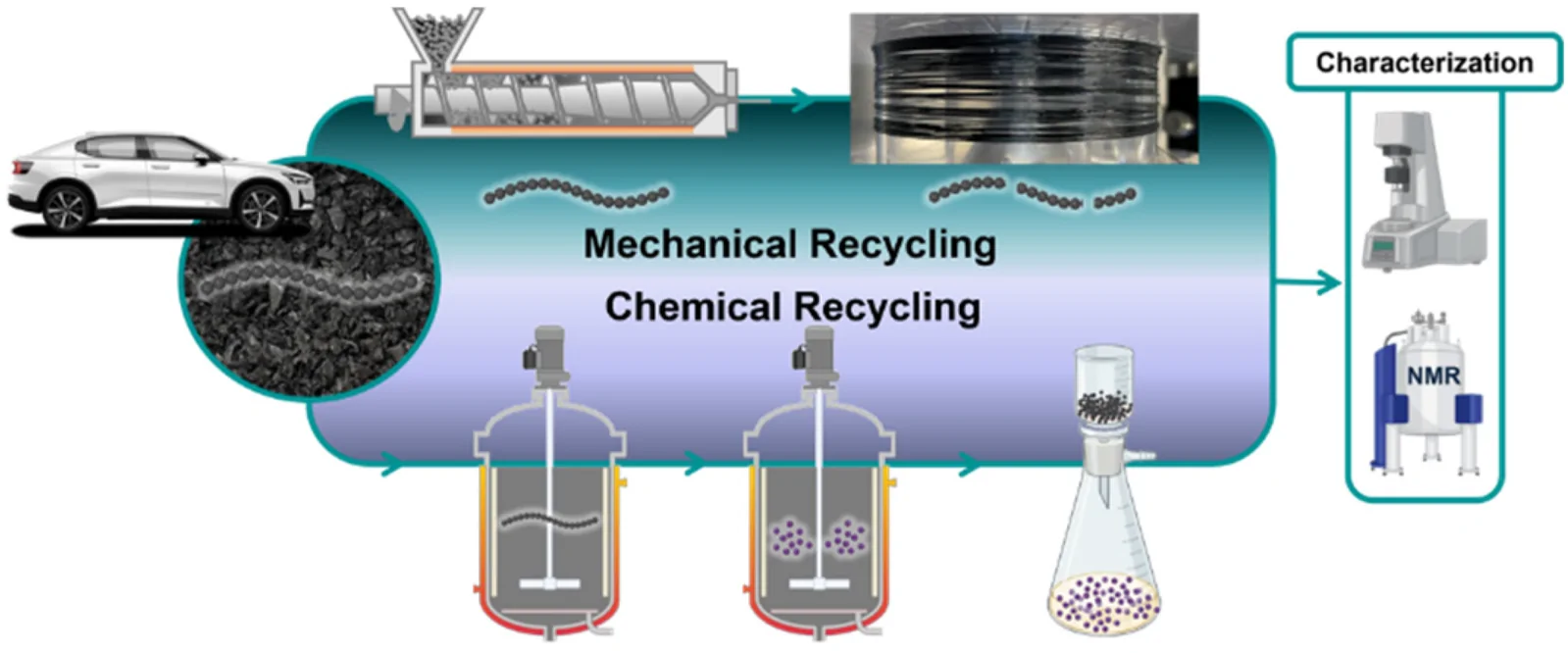

## Introduction

Polymers hold ever-growing applicability as base materials for products in nearly every sector, broadened through the mixing of polymer types and the addition of additives, from water bottles and food packaging to car seats and mattresses.^1^ When considering materials with a single polymeric component and consistent form factor, such as single-use poly(ethylene terephthalate) (PET) water bottles, a range of recycling methods have been developed. Thermomechanical recycling of PET is a mature technology on an industrial scale, with 1.4 million tonnes of recycled PET pellets produced in Europe in 2022 from 2.7 million tonnes of collected PET waste.^2^ Beyond thermomechanical recycling, a myriad of depolymerization techniques have been investigated in the literature for PET, utilizing various catalysts and solvolysis procedures to maximize desired product yields and purity, including hydrolysis, methanolysis, glycolysis, acetolysis, and aminolysis.^3–9^ Of these, hydrolysis and glycolysis produce monomers suitable for direct PET repolymerization.^10^ Several chemical recycling facilities have opened worldwide for PET, with various methods (methanolysis, enzymatic hydrolysis) represented, with several more (glycolysis) announced.^11–14^ However, when expanding the application space to items with variable and mixed form factors, such as automotive parts, the technical feasibility of each of the potential methods will impact what is a reasonable end of life (EoL) solution for each individual item.

Focusing our application space to the automotive industry, the development of engineering polymers in automobiles has significantly increased passenger safety through improved impact resistance,^15^ flame retardancy,^16–18^ airbags and seatbelts.^19,20^ Utilizing polymers for automotive components also grants design flexibility for manufacturers while lightweighting vehicle parts.^19^ With the ease of molding polymers into particular design shapes and with thoughtful selection of materials, manufacturers can improve drive comfort and aesthetics, in pursuit of increased customer satisfaction, brand reputation, and profits. Polymer components are especially cost-effective when compared with alternative traditional parts.^21^ However, each of these plastic components consists of a precise formulation of polymers, rather than a single type, to yield the desired properties. Once these materials reach their end of life, the question about how to recycle them reflects that complexity.

When making EoL decisions for materials, we need to consider not only the most sustainable option, but also that which returns a viable material. As discussed above, monomaterials with simple form factors, such as PET water bottles, have many potential recycling routes available. Thermomechanical recycling plants globally produce bottle-grade PET from bottles, while chemical recycling plants for PET are an expanding industry. While in both cases food contact safe materials can be produced, the recyclate is often applied to other sectors, such as textiles.^22,23^ Beyond bottles, complex form factor items, particularly those in the automotive sector, necessitate more complex recycling pathways, as they must also meet stringent performance standards.^24^ Considering textile interiors, polyesters, specifically PET, have been the most popular automotive fabric choice since the 1990s.^20,25^ As PET is a known readily recyclable polymer, using recycled bottle PET feedstock in automotive textiles is now well established. However, the form factors for PET fabrics are more complex as are their formulations, with additives and backings making them more complex multi-materials than many other PET substrates.^26^ Here we will compare mechanical and chemical recycling routes for a relatively simple mixed material from an electric vehicle – the polyester liner from the front trunk, or frunk. Consisting of layers of woven and non-woven PET sourced from virgin and recycled bottle sources and carbon black colorants generated by the incomplete combustion of hydrocarbons, it is easily removed at EoL, making it feasibly collected with minimal effort. Using a frunk as a feedstock for the production of new frunks or other automotive PET applications would decrease the industrial dependence on PET bottles,^24^ avoiding a cannibalistic economy.

In a techno-economic analysis (TEA) by Uekert, *et al.*, it was determined that thermomechanical recycling currently is the most economical and environmentally benign of the recycling approaches and outperforms virgin polymer production, including for PET.^3^ However, limitations on the maximum number of extrusion cycles before the material properties no longer render mechanical recycling suitable are a lingering concern,^27,28^ and may be exacerbated in non-bottle feedstocks where rebuilding molecular weights may be more challenging. This degradation in properties is attributed to thermo-oxidative or shear induced chain scission or branching, which can be accelerated by contaminants in the PET waste stream.^27,29^ These ultimately affect crystallinity and chain entanglement of the resultant extrudate, which influences the final mechanical properties of the polymer. Successful thermomechanical recycling of a frunk would produce a filament capable of being made into a fiber for a similar application, which can be assessed with rheological methods.

Chemical recycling involves the deconstruction of polymeric materials back to their monomers. A primary advantage of chemical recycling lies in tunability towards certain materials in blends and tolerance to additives. Glycolysis is further favored due to ambient reaction pressures,^30,31^ forming bis(2-hydroxyethyl)terephthalate (BHET), which can be integrated into existing PET synthetic processes. Recent LCA and TEA analyses have shown glycolysis to perform well compared to other depolymerization methods across metrics such as greenhouse gas emissions, energy use, toxicity, water use, and land use.^3,6,32^ Therefore, for this study, glycolysis was chosen as the chemical recycling method. When considering scaling-up glycolysis technology for industrial relevance in car recycling, LCA and TEA indicated that efforts to reduce solvent use and electricity use are key drivers to ensure improved sustainability. Ideally, the results of depolymerizing the frunk would be a glycolysis method tolerant to the additives in the system, yielding BHET which is easily separated from those additives.

While both mechanical extrusion and glycolysis are well established recycling routes for PET bottles, the form factor of the original material plays a role in the pretreatment steps necessary before a feedstock can be processed. Bottles are easily shredded into flakes for extrusion, however textiles have a lower density that may impact the available recycling methods by influencing feeding and increasing surface area for degradation.^33^

In this study, two recycling pathways are compared, mechanical reprocessing and glycolysis (chemical), after different methods of material preparation (Fig. 1). First, the frunk liner is chopped (1), then shredded (2), and then granulated (3), retaining representative material at each stage. These materials were then subjected to either thermomechanical (extrusion) or chemical (glycolysis) recycling and subsequently analyzed by rheology and nuclear magnetic resonance spectroscopy. Here, the two recycling methods are compared on the grounds of the technical feasibility of each method, including pre-processing limitations and feasibility to scale processes, aiming to establish a precedent for comparing not only method sustainability, but object-level feasibility when recommending EoL processes.

**Fig. 1:**
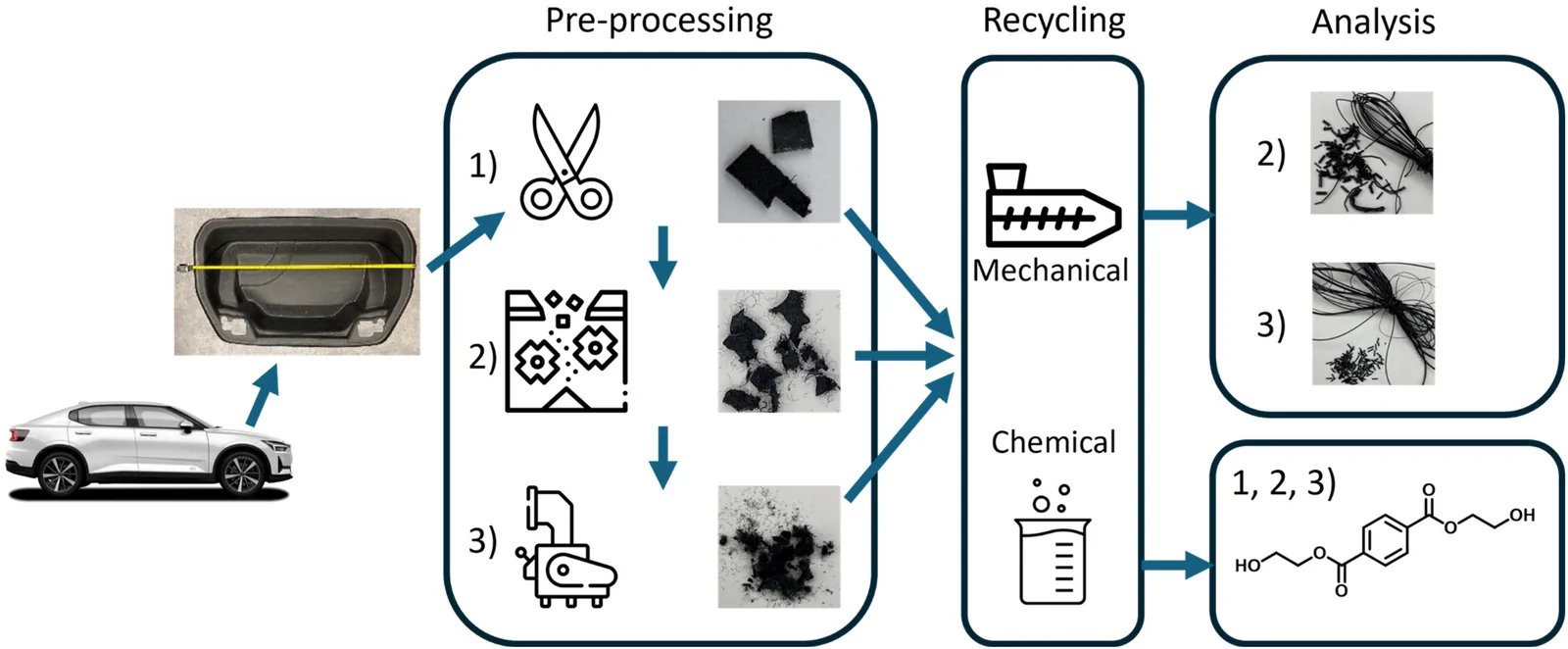
Schematic of comparing recycling methods for the frunk liner. Pre-processing of the liner included (1) cutting into pieces, (2) shredding those pieces, and (3) granulating the shredded material. The shredded and granulated populations were mechanically recycled through an extruder to produce filament and pellets. All three populations were depolymerized by glycolysis to form monomer.

## Materials and methods

### Materials

Automotive-sourced recycled PET (rPET) was obtained in the form of a front trunk (frunk) liner (106 cm × 63.5 cm × 21.2 cm, Fig. S1a) from a Polestar 2 as supplied by Polestar Automotive Holding. The frunk liner is composed of PET fibers sourced from recycled bottles with carbon black added as a colorant. Prior to use, the frunk liner was cut into smaller pieces suitable for a shredder (about 7 cm square). Some pieces were cut further (3 cm square) for testing (frunk-p). The material was then shredded in either a Sustainable Design Studios Shredder Basic or a 3devo Plastic Shredder GP20 (combination shredder/granulator). In the case of the 3devo GP20, two size populations were generated. Material collected from the bottom basket, shredded + granulated (sg), was smaller in size (2.5 mm across) and a bit fluffier (frunk-sg, Fig. S1c), while material that did not pass through the granulator, shredded (s), was slightly larger (5 mm across) and less fluffy (frunk-s, Fig. S1b). The resulting material was dried at 120 °C in a vacuum oven prior to use. Virgin RamaPET (vPET) N1S pellets (Indorama Ventures, 2 mm spheres) were dried under vacuum for a minimum of 12 hours at 120 °C prior to use. Bottle flake (2.5 mm) was obtained by shredding PET water bottles from iPac Packaging Innovations.

Ethylene glycol (EG, Sigma Aldrich), 1,8-diazobicyclo[5.4.0]undec-7-ene (DBU, Sigma Aldrich), diphenyl ether (DPE, Thermo Fisher Scientific), dimethyl sulfoxide (DMSO, Sigma Aldrich), deuterated dimethyl sulfoxide (d_6_-DMSO, Sigma Aldrich), 1,1,1,3,3,3-hexafluoro-2-propanol (HFIP, Fluorochem), and chloroform (CHCl_3_, Merck) were used as received.

### Extrusion

A HAAKE Polylab PTW16-25 twin-screw extruder (L/D = 25) with 7 heating sections, a rod die, and standard Thermo Fisher Scientific screw configuration (3 mixing zones, 4 conveying zones, and 1 discharge zone) was used to process the material. Material was fed into the extruder using a volumetric hopper. Shredded materials were extruded at varied feed rates with a set temperature of 260 °C across 7 heating zones and a screw speed of 65–100 rpm. The Polylab PTW16-25 was fitted with a HAAKE water bath (deionized water) set to 15 °C. The extrudate was drawn through the water bath to produce a filament which was then pelletized on a Thermo Fisher Scientific VariCut pelletizer to a length of 2.5 mm (pelletizer feeder speed of 6 meters per minute).

Single-screw extrusion was performed on a HAAKE Rheomex 19/25 QC lab Single Screw Extruder with 5 heating sections, a rod die, and a 19/25 short metering screw. The extruder was fitted with a HAAKE water bath and Spooler to collect the material. A large diameter steel rod was used to weigh down the low density flood-fed shredded material to boost conveyance.

### Glycolysis

In a generalized procedure, PET (1 equivalent) was mixed in either a 7 mL microwave vial (0.3 g PET), a 500 mL round bottom flask (30 g PET), or a 10 L Syrris Orb Pilot batch reactor (1 kg PET) with EG (5–40 molar eq.) and DBU (0.025–0.1 molar eq.). A detailed description and full table of experiments can be found in the SI section 3 (Table S1, Fig. S4, Fig. S9). The vessels were sealed and degassed for 1 minute with N_2_ prior to stirring at 500 rpm at 180 °C in an oil bath for 1 hour (see SI section 3.3 for specific batch reactor method). The reaction was removed from the heat and allowed to cool to room temperature resulting in precipitation of the depolymerization product (BHET). In the case of the microwave vial, DMSO (1 mL) was added to the reaction vial and vortexed for 4 min to solubilize BHET. An aliquot was taken from this mixture for quantitative nuclear magnetic resonance (NMR) spectroscopy to determine the conversion. In the case of the 30 g and 1 kg batches, deionized water was added to the reaction flask and allowed to cool to 90 °C prior to hot filtration. The resulting filtrate was cooled to room temperature initially and the resulting mixture vacuum filtered again to collect the precipitated BHET, which was subsequently dried at 40 °C in a vacuum oven. Additional BHET was recovered by cooling the mother liquor in a refrigerator overnight.

### Analysis techniques

Melt flow index (MFI) was determined on a Zwick Roell Cflow extrusion plastometer. 7.5 g polymer was added to the barrel and allowed to equilibrate at a given temperature (250–280 °C) for 5 min. A 2.16 kg weight was used to induce flow, with a 15 s cut time for generating samples. Individual samples were weighed and flow rate determined by converting mg per 15 s to g per 10 min.

Small amplitude oscillatory shear (SAOS) rheology was done on a Discovery HR2 rheometer with a 25 mm stainless steel lower plate, an external oven providing heat to 260 °C, and a flow of N_2_ (15 mL min^−1^). 1.0–1.5 g of sample (either pellets or shredded/granulated material) was loaded onto the lower plate at temperature, and the upper plate lowered to a trim gap of 1050 μm. The trim resulted in a loss of up to 0.60 g of product, leaving 0.4–0.7 mg on the plate. A test gap of 1000 μm was used. An amplitude sweep (1–100% strain, 1 rad s^−1^ frequency) was performed to test the viscoelastic regime. A frequency test (0.1–600 rad s^−1^) was performed at 1% strain and collecting 10 points per decade, and the resulting data was plotted as phase angle *vs*. complex modulus (MPa) in a van Gurp-Palmen plot to investigate the morphology of the polymer.

Quantitative NMR spectroscopy (qNMR) was performed on a Bruker Avance III 400 MHz spectrometer. The conversion of PET to BHET was calculated from the depolymerization reactions following eqn (S1)–(S4) of the SI. Diphenyl ether (DPE) was added as the standard, and stock solutions were prepared at a concentration of 0.02 g mL^−1^ in DMSO-d_6_. To an NMR tube, 0.016 g of solid sample or 0.2 mL reaction mixture in DMSO was added to 1 mL DPE stock. These were then analyzed by ^1^H NMR and the ratio of the integrals of the peaks of interest (BHET: 8.12 ppm, DPE: 7.13 ppm) used to calculate the conversion from PET to BHET (Fig. S6–S8). The mass of PET was assumed to be the total mass of the frunk sample, as TGA and mass recovery results revealed that carbon black is present at about 2 wt% in the samples (Fig. S15 and S16).

## Results and discussion

The frunk chosen for this study was removed post-production from a Polestar 2. The frunk was approximately 106 cm × 63.5 cm × 21.2 cm, primarily composed of recycled PET, and its coloration was derived from carbon black. For both mechanical and chemical recycling methods, size reduction was necessary to enable processing. Pieces of about 7 cm square were cut from the whole and fed into a combination shredder–granulator, which resulted in two size populations, frunk that was shredded only (frunk-s) and frunk that was both shredded and granulated (frunk-sg), as shown in Fig. S1. These materials were subsequently compared across mechanical and chemical methods.

### Thermomechanical recycling

To determine the optimal extrusion temperature condition, the material was first analyzed by melt flow experiments (Fig. S2). At 280 °C, the standard analysis temperature for PET, the material flowed too quickly out of the instrument, such that an MFI was unable to be accurately calculated. Therefore, the temperature was lowered to a range of 250–260 °C, and experiments performed in that range. It was found that 260 °C yielded an MFI of around 60 g per 10 min, which is sufficient for extrusion.

Twin screw extrusion served as the first extrusion model for the thermomechanical recycling of the material. To identify an appropriate feeding rate from the volumetric feeder, the set feed rate was varied and the actual rate determined at that set feed rate by weighing the material that exited the feeder over 60 s (Fig. 2a, detailed description in SI). After optimization, a set feed rate of 50, which on average corresponded to 163 ± 11 g h^−1^ for frunk-s or 28 ± 1 g h^−1^ for frunk-sg, was chosen. Further optimization exceeded the scope of this work.

**Fig. 2:**
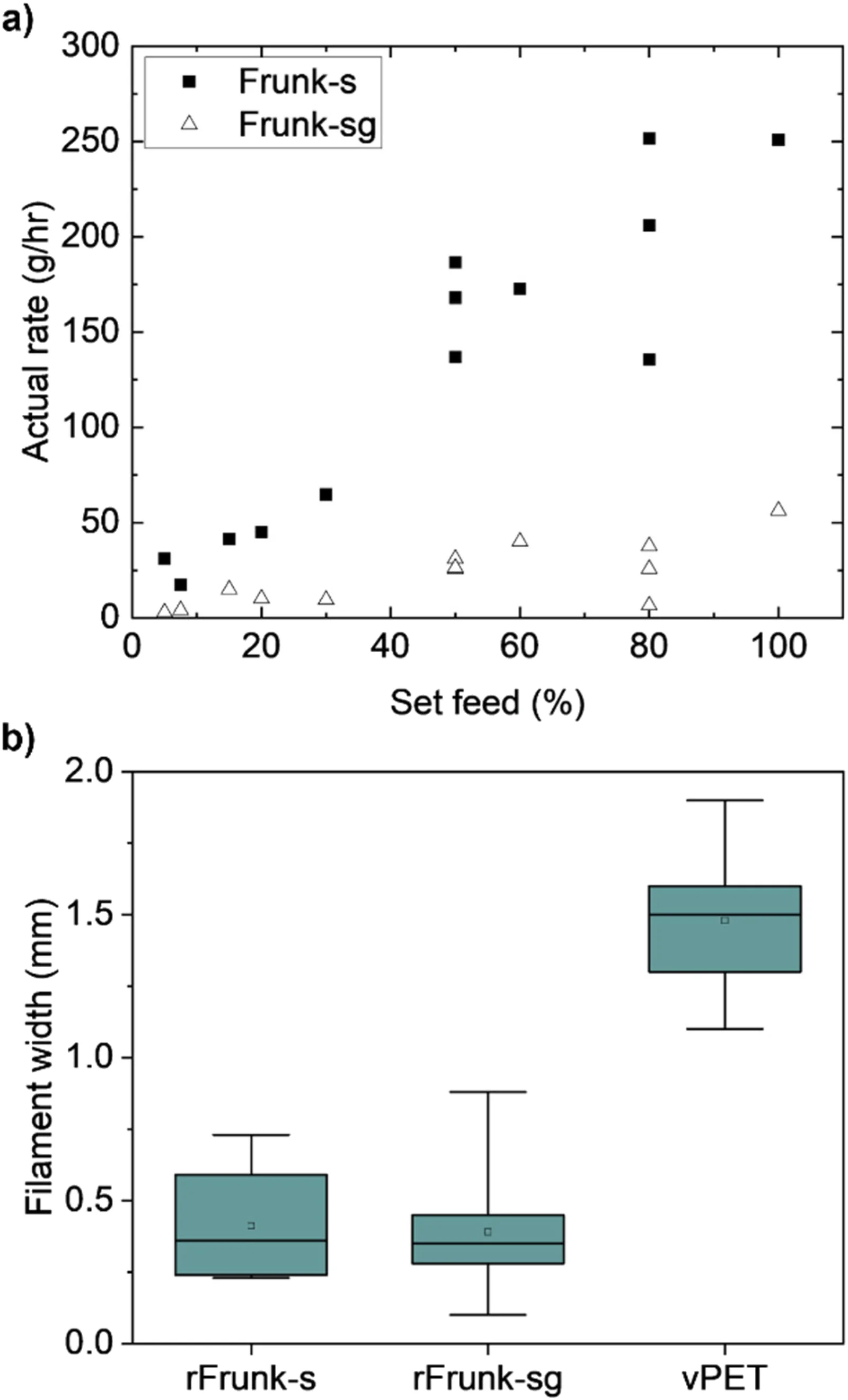
Thermomechanical recycling of PET sourced from the frunk trunk (frunk) of an EV. (a) Feed rate of frunk material at varying set feeding rate on the extruder for both frunk-s and frunk-sg, highlighting inconsistency in feeding. As a comparison, virgin bottle grade PET pellets feed at 800 g h^−1^ at a set feeding rate of 7.5%. (b) Distribution of widths for filaments formed when extruding frunk-s (15 measurements), frunk-sg (15 measurements), and vPET (5 measurements). The box is the 25^th^ to 75^th^ percentiles, with the whiskers from the min to max values.

After drying, 50 g of frunk-s or frunk-sg were added to the gravimetric feeder. As the material left the feeder, it could not be caught by the extruder screws to enter the barrel and instead built up on top of the screws (SI Video). Therefore, the amount of material being dispensed was not the same as what was entering the extruder, contrary to a conventional extrusion set up, which resulted in an inconsistent filament (Fig. S1b and c). The extrudate did not improve through change of screw configurations, weighted feeding or pre-drying variations and was collected with a similar quality independent of the extrusion conditions. The collected filament was a shiny black color and was narrow, with a diameter of about 0.4 mm from both frunk-s (postextrusion filament rFrunk-s) and frunk-sg (rFrunk-sg) (Fig. 2b). From the 50 g material added to the feeder, less than 5 g of extrudate was collected over the extrusion, with loss due to material failing to feed as well as residue in the feeder. Further optimization, or upscaling would likely reduce the amount lost in the hopper and thereby the yield of the process. However, since the scope of this project was rather a comparative feasibility study, this successful transformation served as a proof of concept. In order to assess the performance of the extrudate, the rFrunk-sg was pelletized for rheological analysis.

To understand the impact of the extrusion on the frunk material, SAOS frequency sweeps were performed. Based on our previously reported method,^34,35^ experiments were run at 260 °C and in an N_2_ atmosphere to give van Gurp-Palmen (vGP) plots of phase angle (*δ*) *versus* complex modulus (G*) (Fig. 3).^34^ The direction of the shift in various parts of the curve from before processing (purple squares, frunk-sg) to post-processing (green circles, rFrunk-sg) indicated which type of degradation occurred during recycling.^34–36^ The minimum in *δ* around 10^−3^ MPa in frunk-sg indicated the presence of long chain branches, consistent with recycled PET, from which the frunk liner was made.^34,37^ While virgin PET contains isophthalic acid units to assist with processing, often those linkages are the first to break when reprocessing.^38,39^ When comparing rFrunk-sg with frunk-sg, the curves shifted towards lower G* and flattened with respect to *δ* implying severe degradation because of the thermomechanical recycling process, with the flattening indicating a shift from a branched structure towards a system that had experienced chain scission. As the starting frunk-s material was already on the low end of fiber-grade PET (*M*_w_ = 52.4 ± 0.1 kDa, Fig. S3), further chain scission when generating rFrunk-sg indicates that the material could not be reused as fibers.^40,41^

**Fig. 3:**
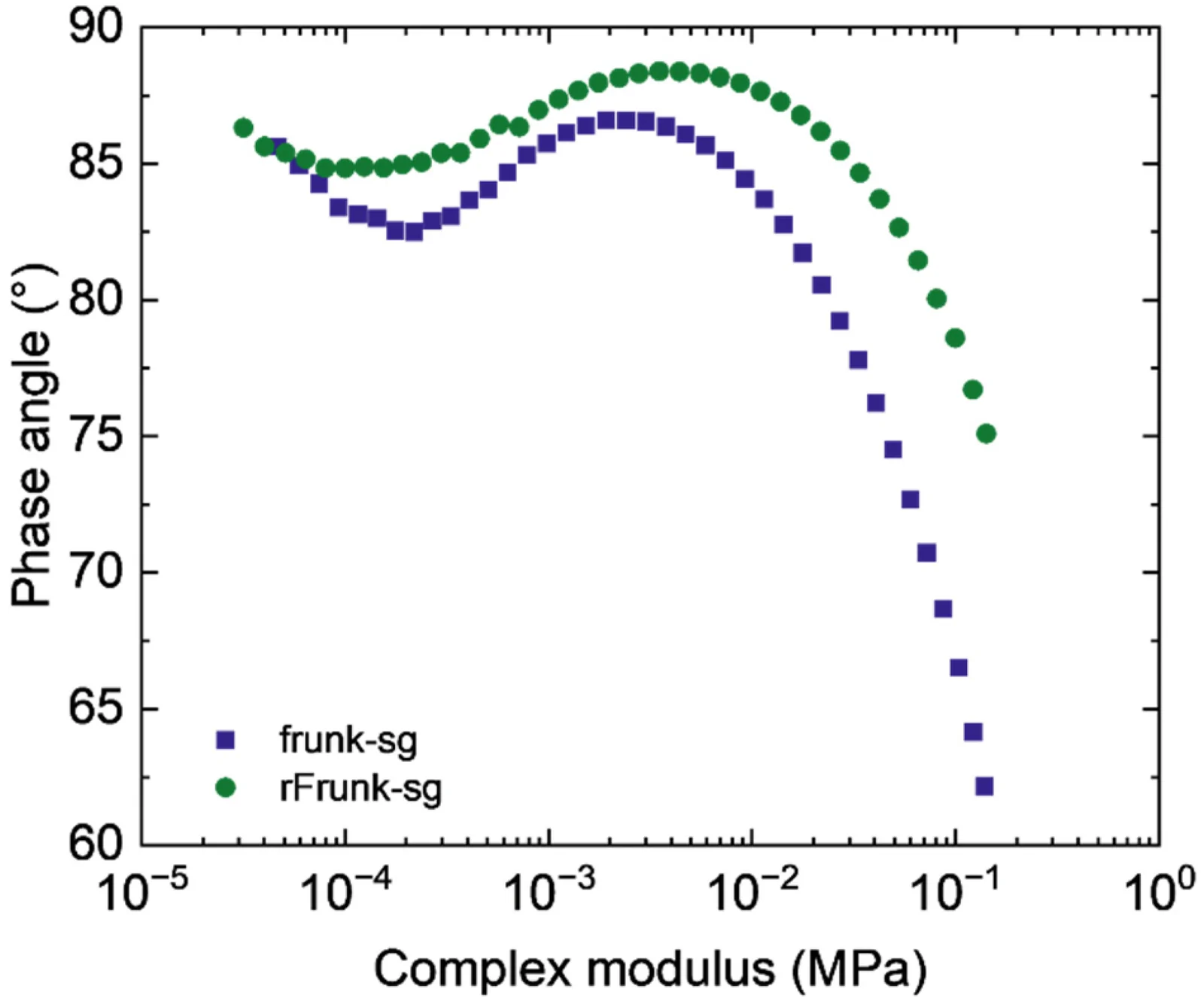
van Gurp-Palmen plot frunk-sg (purple) and rFrunk-sg (green). The change in shape indicates chain scission and a loss of chain branching occurred during processing, further highlighted by the shift to lower G*, consistent with decreasing molar mass.

Single-screw extrusion was also explored as a possibility, with a flood feeder rather than a starve feeder. However, the low density of the frunk-s did not allow for material flow into the single-screw to be significantly more effective than the twin-screw for filament/pellet production, even when the material was weighed down to encourage feeding.

Therefore, it was determined that thermomechanical recycling was challenging for the frunk material, especially in comparison to bottle feedstock normally used in automotive rPET fiber production. While developing pretreatment methods or chain extension strategies could increase the density of the feed, facilitate extrusion, and improve properties for the product, these treatments could influence industrial resistance to uptake. The presence of additives, including carbon black, additionally limits further application of rFrunk-s and rFrunk-sg.

### Chemical recycling

In chemical recycling, the polymer is deconstructed into monomers prone to repolymerization into virgin-grade materials. Separating the formed small molecule from the used additives then allows either the recycling of the additives or adding new additives to prepare materials with different properties that can be used in any given application. Here, glycolysis was used since it yields BHET as a product compatible with industrial polymerization of PET (Fig. 4a). In a first step the reaction conditions as well as form factor of materials were explored. DBU was chosen as a catalyst due to its success in previous studies on non-bottle PET sources.^7,42^ After the reaction, qNMR was performed on the reaction mixture to determine conversion with diphenyl ether as an internal standard at 0.2 g in 10 mL DMSO, and assuming all sample mass added to the reaction was PET (SI eqn (S1)–(S4)). Based on TGA experiments, and backed up by residual recovery, the average loading of carbon black was calculated to be 2% in the frunk by mass (SI sections 3.4 and 4), suggesting that the obtained yield by this method was only slightly overestimated. However, since the amount of carbon black could not be quantified, this uncertainty was propagated into the conversion calculations.

**Fig. 4:**
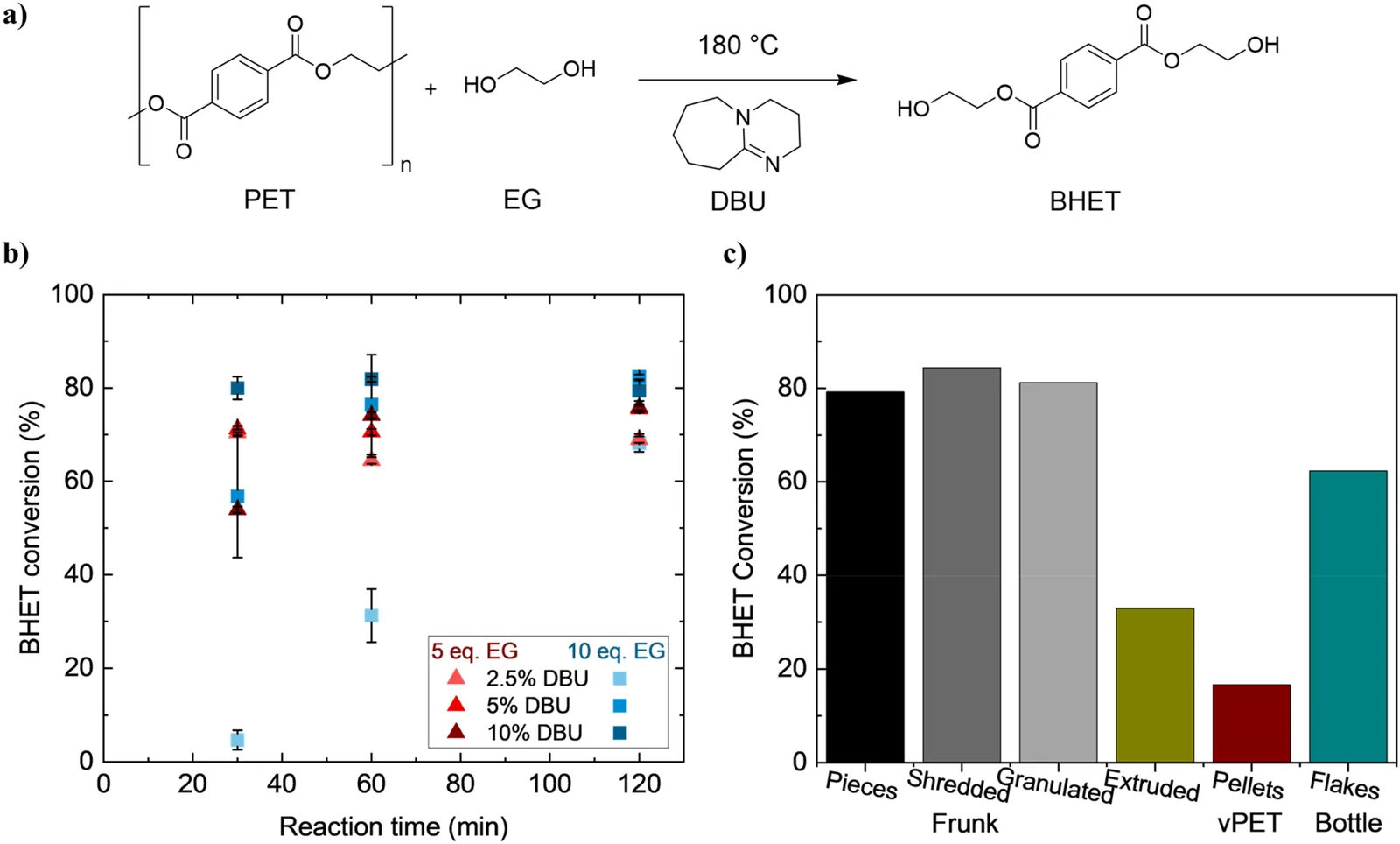
(a) Scheme of frunk-s depolymerization by glycolysis with DBU catalyst. (b) DBU catalyst loading variation (2.5, 5, 10 mol%) with either 5 eq. EG (red) or 10 eq. EG (blue). (c) Influence of starting material shape on BHET conversion, 5 min reaction time, all samples with 10 mol% catalyst and 10 eq. EG. Conversion determined by quantitative NMR analysis.

To optimize the reaction, a starting mass of 0.3 g frunk-s was used, while the concentrations of DBU and EG were varied. For DBU concentration, 2.5, 5, and 10 mol% catalyst loading was tested on frunk-s at 5 and 10 equivalents of EG, sampling at 30, 60, and 120 min (Fig. 4b). With increasing DBU concentration, the time to full conversion was faster. A higher content of EG (10 eq.) led to a stronger DBU concentration dependence, with 30 min of reaction time sufficient for 10% DBU to reach 80% conversion. In some cases, longer reaction times led to slightly lower BHET yields, which can be attributed to the formation of oligomers.^43,44^ As the 10% DBU and 10 eq. EG gave the maximum conversion in the shortest time, all further experiments were conducted at these conditions. However, independent of the reaction conditions, and even after 2 h of reaction time, only a maximum conversion of roughly 80% could be obtained. For the samples with a content of 10% DBU and 10 eq. EG, this maximum conversion could be obtained within 30 min.

The influence of PET form factor on glycolysis was investigated by comparing various starting geometries of frunk and rFrunk-sg extrudate along with bottle grade PET pellets and bottle flake (Fig. 4c, Table S2). The reactions were run for 5 minutes to best see a trend in behavior. Frunk-s, frunk-sg, and frunk-p all reached 80% conversion in that time, as determined by qNMR. rFrunk-sg extrudate only reached 33% conversion. vPET and bottle flake reached 17 and 60% conversion within the same time frame, all with the same starting mass of PET. Due to the high density, low surface area, and limited swelling of the PET-pellets, both virgin and frunk extrudate, their conversion was much slower and hindered by diffusion. This indicated that the form factor of the material matters, with the fibrous nature of the frunk improving reagent access to the PET, and that none of the additives in the frunk were hindering depolymerization. While molar mass and crystallinity also have an impact on glycolysis rates, the decrease in conversion of rFrunk-sg indicated that form factor plays a major role. On the contrary, for dense materials, an additional swelling or shredding process would be suggested.

To probe the industrial relevance of frunk glycolysis, the reaction was scaled up to both 100-fold (30 g) and to 1 kg of frunk liner. For both reactions, 10 eq. of EG at 10% DBU were used, consistent with the kinetic study (Fig. 4b). The shredded material was either placed into a 50 mL round bottom (30 g scale, Fig. S10) or into a 10 L batch reactor (1 kg scale, Fig. 5a) along with EG and DBU, generating a total volume of 3 L for the 1 kg scale. After 2 hours of stirring at 180 °C, the material had disintegrated, visually leaving carbon black particles floating in an ethylene glycol/BHET solution (Fig. 5b). An aliquot was taken of the crude mixture, and the conversion determined to be 62.6% for the 1 kg scale by qNMR (Fig. S7). Upon cooling, deionized water was added to dissolve the BHET, and the system filtered hot (90 °C) to keep the BHET in solution. After hot filtration, a liquid fraction of BHET and EG in deionized water (Fig. 5c) was clearly separated from the solid fraction of additives (Fig. 5d). BHET was recovered by subsequent cold filtration and water washes to remove the ethylene glycol (Fig. 5e). On the 30 g scale, 60% molar yield BHET with respect to frunk-s added (assuming 100% of the frunk was PET, and that 100% of the moles of PET could be converted to BHET) was recovered, which increased to 90% upon repeating the washing and filtration steps. From the 1 kg scale, 626 g of BHET were recovered, a 47% yield. When correcting for the frunk liner containing 2 wt% additives (SI sections 6 and 7), and the reaction could give a theoretical yield of all 980 g of PET converting to BHET, this gives a 48% yield of BHET, which is within the expected error of the synthesis technique. In both cases, the decrease in yield occurs during coprecipitation of BHET with carbon black in the product filtration and incomplete recrystallization. After hot filtration, some BHET crystals can be seen forming in the carbon black residue left on the filter paper (Fig. S12). Additionally, further cooling of the mother liquor led to further crystallization of BHET, indicating that the purification cold water washes also dissolved some of the product. Further optimization might increase the yield accordingly, but was beyond the scope of this work. The recovered BHET was white crystals, with little discoloration from any residual carbon black material (Fig. 4e). They were analyzed by 2D NMR and HPLC (Fig. S13 and S14), and found to match commercial BHET. This material could then be repolymerized to PET without concern for additive contamination in the new synthesized polymer.

**Fig. 5:**
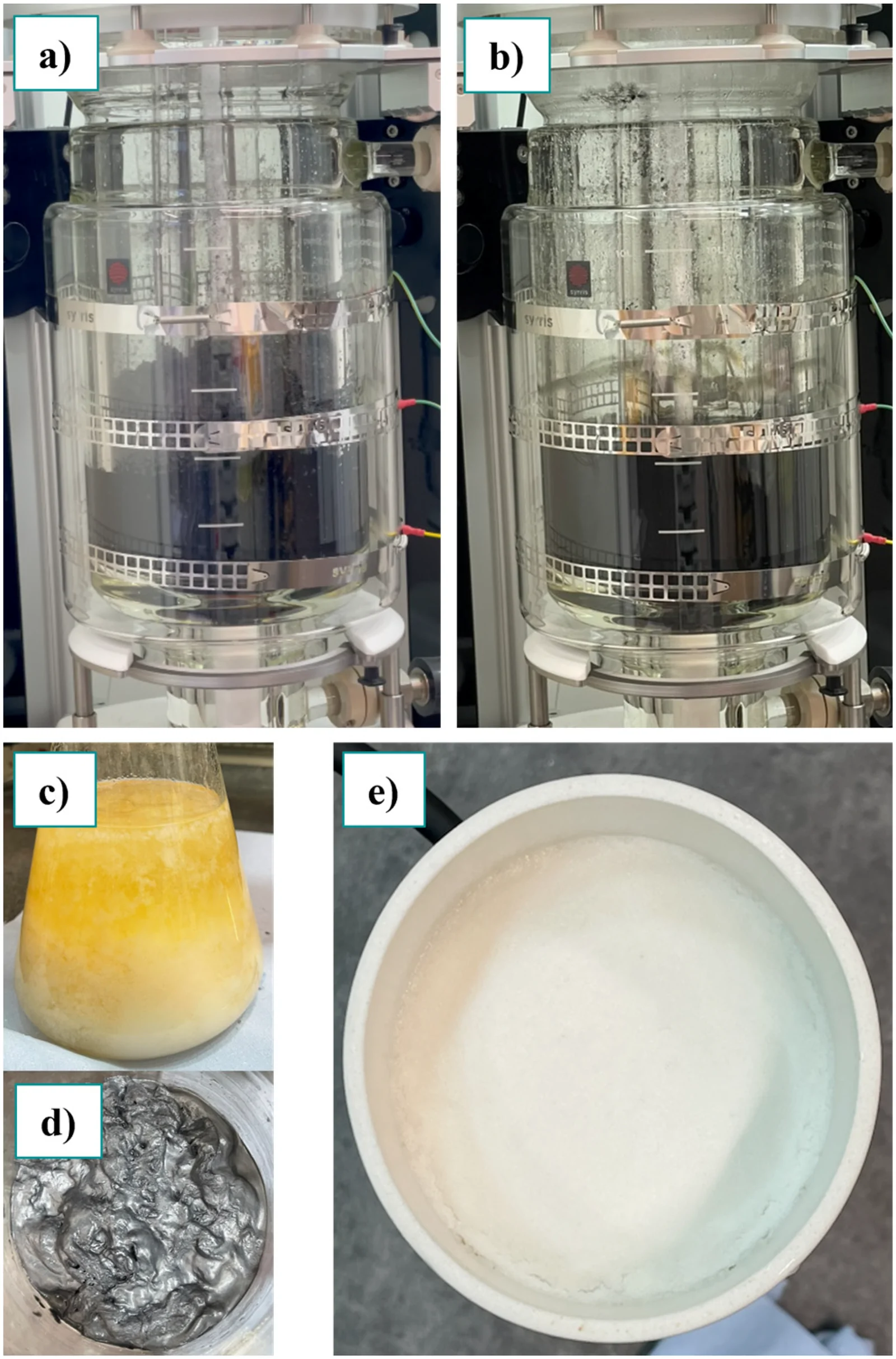
Scale up of frunk glycolysis to 10 L reactor. (a) Reactor containing an entire shredded frunk (1 kg) along with EG (3 L) and DBU catalyst (79 mL) prior to the start of the reaction. (b) Reactor after 2 hours of stirring at 180 °C. (c) BHET solution after filtration. (d) Remaining material separated from BHET. (e) Recovered BHET (626 g), a 47% yield.

To test if the frunk could be added to a larger reactor as pieces rather than fluff, frunk-p was depolymerized at the 30 g scale, and the same water workup followed. The resulting yield was 50% after recrystallization, indicating that the density of the chunks was low enough to allow for sufficient penetration of the reaction mixture into the polymer matrix. Intense shredding is therefore not required and the previously observed diffusion limitation in the pellets in Fig. 4b was not observed. One could imagine that on an industrial scale frunks need not be shredded at all before introduction to a large batch reactor. Overall, the reaction was found to be scalable from 0.3 g to 1 kg and generated a product that could be fed into a reactor to synthesize new PET.

With chemical recycling successfully scaled up to 1 kg, and small-scale reaction conditions reaching 80% conversion in less than half the time of bottle-grade feedstocks, the form factor that made thermomechanical recycling so difficult successfully suggests chemical recycling to be the favored recycling technique. Considering that the used PET stemmed already from processed bottle-grade PET, chemical recycling and repolymerization into new PET bottles appears to be a sensible pathway to close the loop in terms of a circular economy.

## Conclusion

The recyclability of a non-woven fibrous PET car component was tested through mechanical extrusion and chemical glycolysis. Thermomechanical recycling of the frunk PET was hindered by inconsistent feeding which did not improve during optimization efforts, leading to inconsistent filament width, and significant shear-based damage producing low-quality extrudate, as evidenced by small amplitude oscillatory shear rheology. Glycolysis, on the other hand, could easily penetrate the low-density fibrous material, leading to faster depolymerizations than either bottle flake or virgin, crystalline pellets. With optimized DBU catalyst and ethylene glycol loadings, the depolymerization was successful on both a 30 g and 1 kg scale, yielding 50–90% BHET after washing and recovery. The resulting BHET was able to be separated from the carbon black and other additives and therefore would be polymerizable on an industrial scale, with the possibility of further improving yields by optimizing downstream separation processes.

While this comparison was successful for the frunk material, we also acknowledge that we were working with a PET compounded with additives that were agnostic towards the glycolysis process, which gave us a clear preferred recycling method. Other mixed materials may not yield such a straightforward comparison, with some mixed polymers potentially requiring swelling (such as in PC/ABS). Looking towards the future, mixtures of PET sources along with additive and contaminant challenges may skew the results towards a need for more extensive pre-processing, as well as downstream processing, methods.

## Author contributions

Claire L. Seitzinger: conceptualization, investigation, methodology, writing – original draft. Lynn Anderson: conceptualization, methodology, writing – original draft. Fabian Mehner: investigation, writing – original draft, Ciaran W. Lahive: conceptualization, writing – review & editing, Helena Simmons: resources, conceptualization, supervision, funding acquisition. Michael P. Shaver: conceptualization, supervision, funding acquisition, writing – review & editing.

## Conflicts of interest

The authors declare no conflicts of interest.

## Supplementary Material

LP-OLF-D6LP00330C-s001

LP-OLF-D6LP00330C-s002

## Data Availability

The data that support the findings of this study are available in the supplementary information (SI) of this article. Supplementary information is available and includes further experimental details on mechanical and chemical recycling and process optimisation. See DOI: https://doi.org/10.1039/d6lp00330c.
