# Supplementary material for: Up/down frunk: comparing recycling methods for non-woven poly(ethylene terephthalate) from an electric vehicle
Source: RSC Appl Polym. 2026 Oct 1. Online ahead of print. doi: 10.1039/d6lp00330c (PMC13640944; doi:10.1039/d6lp00330c)
Supplement: Supplementary file 1 [file LP-OLF-D6LP00330C-s001.pdf]

Supplementary Information (SI) for RSC Applied Polymers.
This journal is © The Royal Society of Chemistry

Supporting Information for

Up/Down Frunk: Comparing Recycling Methods for Non-Woven Poly(ethylene terephthalate) from an Electric Vehicle

Claire L. Seitzinger [1], Lynn Anderson [1], Fabian Mehner[1], Helena Simmons[2], Ciaran W. Lahive [1], Michael P. Shaver [1,*]

[1] Sustainable Materials Innovation Hub, Department of Materials, Henry Royce Institute, University of Manchester, M13 9PL, United Kingdom

[2] Polestar Automotive UK Ltd, Envisage, Unit F, Coventry, UK

* michael.shaver@manchester.ac.uk

Table of Contents

1 Table of Contents 1
2 Mechanical recycling 2
2.1 Optimization of extrusion parameters 2
2.1.1 Extrusion sample photos 2
2.1.2 Melt flow index 3
2.1.3 Size exclusion chromatography 3
3 Glycolysis 4
3.1 NMR spectroscopy 6
3.2 Reaction optimization 10
3.3 Detailed scaled-up glycolysis procedures 10
3.4 Quantifying carbon black additives 15
4 Thermal properties 16
5 Effect of sample density on PET conversion 17

1 Mechanical recycling

1.1 Optimization of extrusion parameters

For vPET as a feedstock, set feeds of 2%, 5%, and 7.5% result in feeds of 110, 570, 830 g/h. However, when both frunk-s and frunk-sg PET were tested, significant variability was observed between the set and actual feeding rates (Figure 2a in the main text). Triplicate measurements at 80% set feed rate revealed a range of over 100 g/h measured rate for frunk-s. For frunk-sg, one of the measured rates was at 6.6 g/h, a near-zero result. Triplicate measurements at 50% feeding rate were more consistent (163 ± 12 g/h for frunk-s and 28 ± 1 g/h for frunk-sg), with less variability. Therefore, 50% feeding rate was chosen going forward.

Initially, a screw speed of 100 rpm was targeted to facilitate uptake of the material by the screws and convey it through quickly. However, both the frunk-s and frunk-sg failed to be taken up by the screws and instead sat atop the screws for variable times upwards of 20 seconds per clump, leading to a buildup of material (SI Video). Decreasing the screw speed to 70 rpm to facilitate more material uptake by the screws did not increase consistency of material uptake. A slow haul off speed was required to generate pellets that were wide enough for the pelletizer to adequately cut into pieces. The filament from frunk-s (rFrunk-s) had an average width of 0.41 ± 0.04 mm (15 measurements), and frunk-sg (rFrunk-sg) averaged 0.39 ± 0.05 mm (15 measurements). In contrast, at similar screw speeds and cut times, processing vPET results in an average width of 1.5 ± 0.1 mm (5 measurements).

Extrusion sample photos

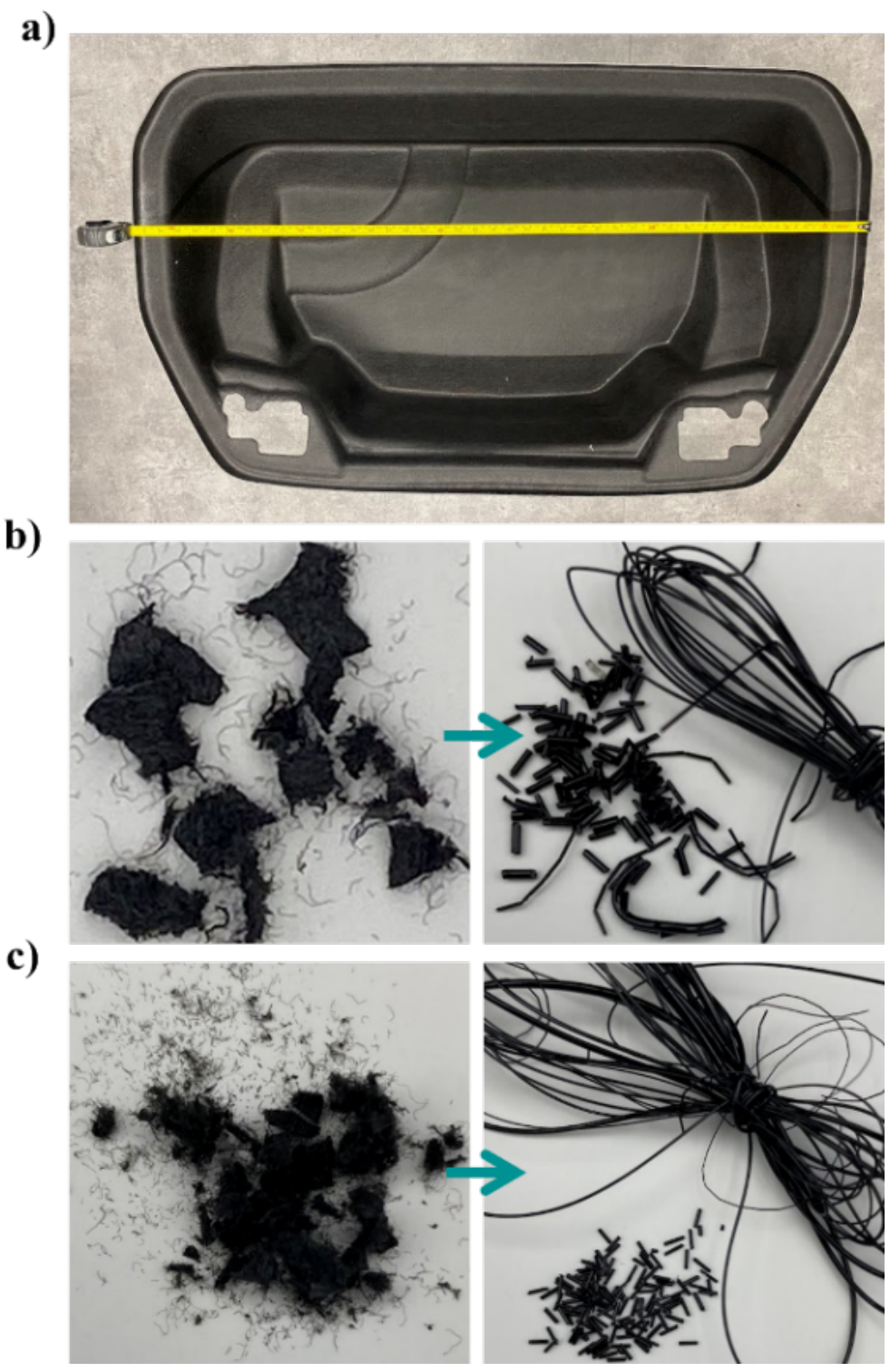

Figure S1. Photos of the a) whole frunk, b) shredded frunk (frunk-s) and extrudate rFrunk-s and c) shredded+granulated frunk (frunk-sg) and extrudate rFrunk-sg. The extrudate formed was highly variable in size.

1.2 Melt flow index

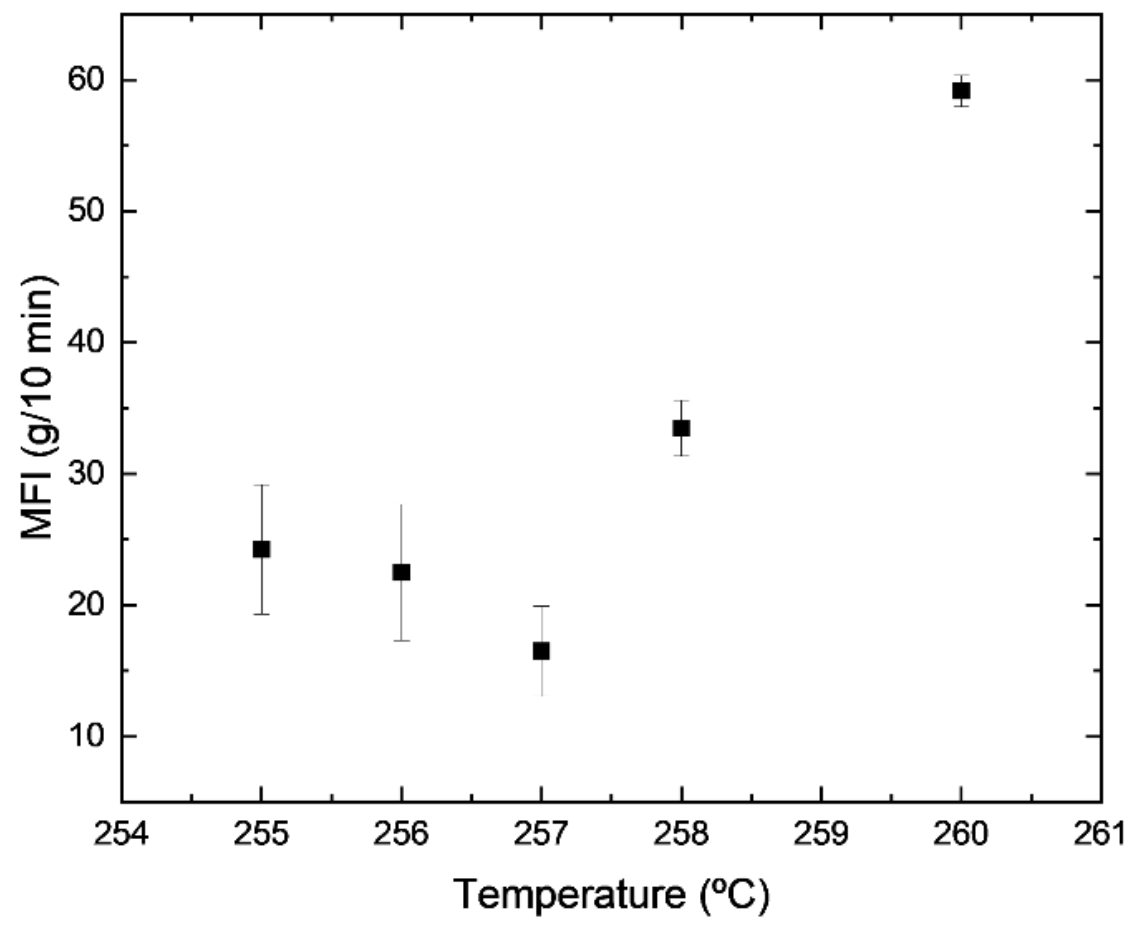

Figure S2. Melt flow index (MFI, g/10 min) at 5 different temperatures for shredded frunk. Each sample had a 5 min residence time, a 15 s cut time, a 2.16 kg weight to encourage flow, and 7.5 g of sample was added to the machine for the tests.

1.3 Size exclusion chromatography

To determine the molar mass of the substrates used and to compare the starting size to literature grading metrics, size exclusion chromatography (SEC) was performed on an Agilent 1260 Infinity II LC system with an Agilent guard column (PLGel 5 µM, 50 × 7.5 mm) and two Agilent Mixed-C columns (PLGel 5 µM, 300 × 7.5 mm). Frunk-s (4 mg) was dissolved in hexafluoroisopropanol (HFIP, 120 µL) by stirring at 40 ºC for 20 minutes. 1880 µL of chloroform (HPLC grade) was added and allowed to stir for 3 hours. The samples were then filtered through 0.2 µm PTFE filters before placing them in septum vials for SEC analysis. The detector was a UV/vis at 300 nm, and molar mass was determined by Universal Calibration against linear polystyrene standards ($162 – 2.4 \times 105$ g mol^{-1}). Six repeats were done of the frunk-s material, with the average M_n was 23.8 ± 0.3 kg/mol and M_w 52.4 ± 0.1 kg/mol, which falls in the range of fibre-grade PET.[1]

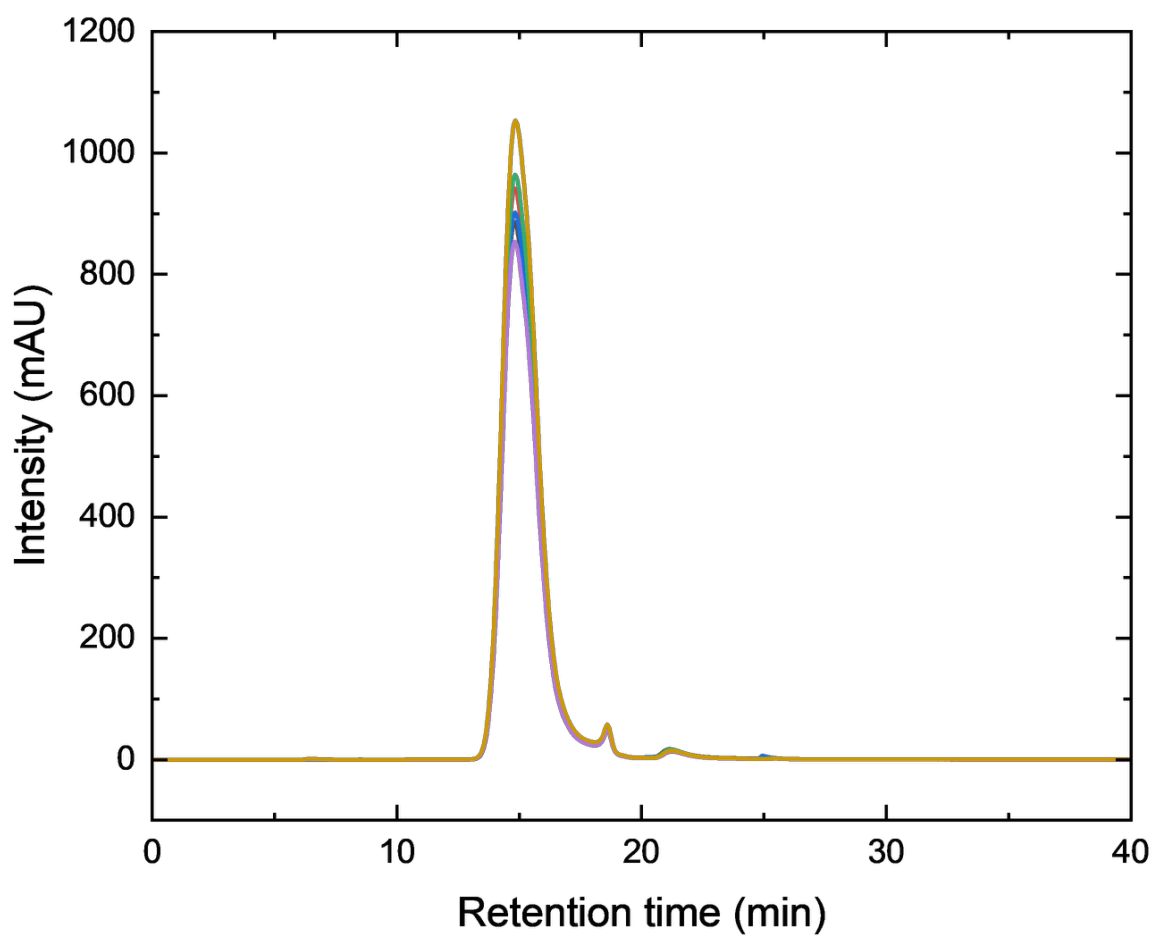

Figure S3. Size exclusion chromatograph of frunk-s over six repeats.

2 Glycolysis

Detailed 0.3 g scale glycolysis procedure

Frunk PET, vPET, or bottle flake (0.3g, 0.0016 mol) was mixed in a 7 mL microwave vial with ethylene glycol (EG, 0.43 mL, 0.0076 mol, 5.01 eq.) and 1,8-Diazabicyclo[5.4.0]undec-7-ene (DBU, 0.023 mL, 0.00015 mol, 0.1 eq.). For the reaction optimization, 0.050 and 0.025 eq. of DBU were also used, as well as 10 eq. EG.

The vial was sealed and the sample was degassed with N_2 for 1 min prior to stirring at 500 rpm at 180 ºC in an oil bath for a varied time (5 min to 17 h). Upon heating, the soaked frunk pieces collapsed and formed a black liquid. The reaction was removed from the heat and allowed to cool to room temperature resulting in precipitation of the depolymerization product (BHET). In the case of frunk material, the formation of white BHET crystals were observed among carbon black residue. DMSO (1-2 mL) was added to the reaction vial and stirred for up to 15 min to solubilize BHET. An aliquot was taken from this mixture for quantitative nuclear magnetic resonance (NMR) spectroscopy to determine the conversion.

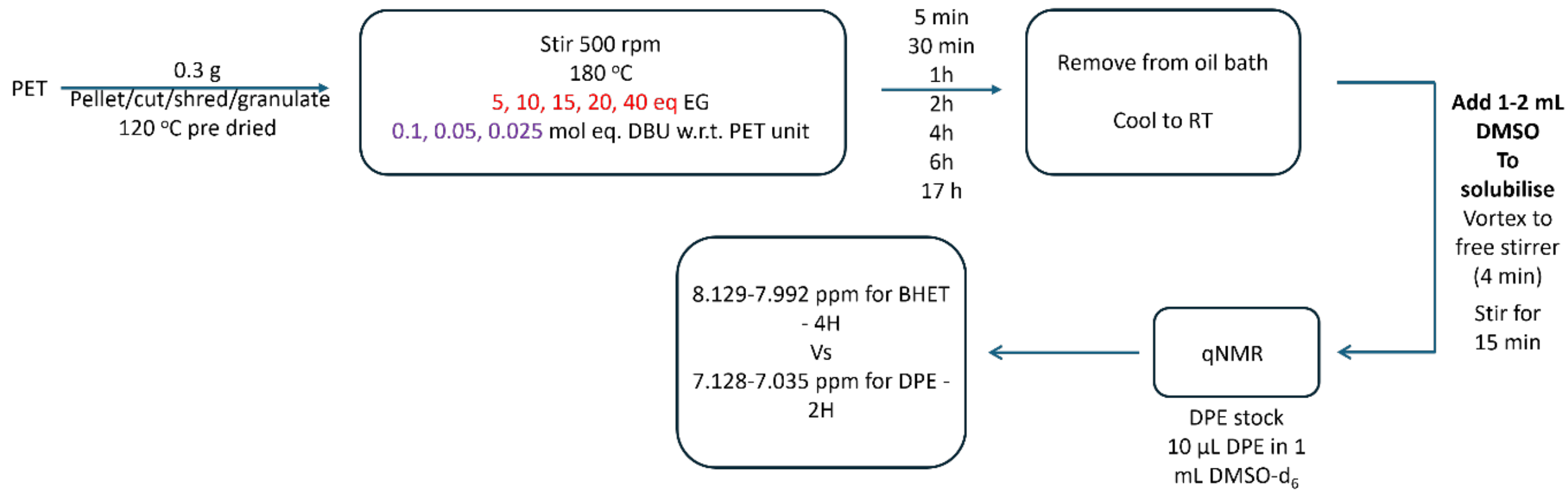

Figure S4. Schematic of reaction steps for 0.3 g scale reactions.

Table S1. Table of reaction details.

Material	Form	PET (g)	Ethylene glycol (mL)	EG equiv	DBU (mL)	DBU equiv	Time (min)
Frunk	shred	0.3	0.43	5	0.023	0.1	30-1020
Frunk	shred	0.3	0.87	10	0.023	0.1	30-1020
Frunk	shred	0.3	1.75	20	0.023	0.1	30-1020
Frunk	shred	0.3	3.49	40	0.023	0.1	30-1020
Frunk	shred	0.3	0.43	5	0.023	0.1	120
Frunk	shred	0.3	0.43	5	0.023	0.1	240
Frunk	granulate	0.3	0.87	10	0.023	0.1	5
Frunk	shred	0.3	0.87	10	0.023	0.1	5
Frunk	chunk	0.3	0.87	10	0.023	0.1	5
Frunk	Extrudate	0.3	0.87	10	0.023	0.1	5
Frunk	shred	0.3	0.43	5	0.023	0.1	30
Frunk	shred	0.3	0.43	5	0.0117	0.05	30-120
Frunk	shred	0.3	0.43	5	0.0058	0.025	30-120
vPET	Bottle flakes	0.3	0.87	10	0.023	0.1	5
vPET	pellets	0.3	0.87	10	0.023	0.1	5
Frunk	shred	30	87.3	10	2.33	0.1	120
Frunk	shred	30	43	5	2.3	0.1	120
Frunk	chunks	30	43	5	2.3	0.1	120
Frunk	shred	30	43	5	2.3	0.1	120
Frunk	shred	1000	2950	10	79	0.1	120

NMR spectroscopy

Quantification by NMR was done by first preparing a stock solution of DMSO-d6 with diphenyl ether (DPE) as an internal standard. 0.03 g of DPE was dissolved in 3 mL DMSO-d_6. 1 or 2 mL of DMSO were added to the reaction mixture to dissolve the BHET. 0.4 mL of stock solution was then mixed with 0.1 mL of the reaction mixture in an NMR tube. The conversion was calculated as follows:

First, the theoretical moles of BHET ($n_{\mathrm{BHET,theo}}$) was calculated from the mass of PET, assuming all PET was transformed into BHET. Thus, the mass of PET (m_{PET}) was divided by the molar mass of a PET repeat unit ($M_{\mathrm{repeat\ unit}}$).

$$n_{BHET,theo} = \frac{m_{PET}}{M_{repeat\ unit}} \qquad \text{Equation 1}$$

Then, in Equation 2, the amount of substance of BHET ($n_{\mathrm{BHET,rxn}}$) was calculated using the DPE internal standard. The known amount of substance for the standard in the reaction (n_{DPE}) was divided by the integral of the peak at 7.08 ppm (I_{DPE}), with N_{DPE} as the number of protons in the integral (in this case 2), and then multiplied by the integral of the BHET (I_{BHET}) at 8.13 ppm, with N_{BHET} as the number of protons in the integral (in this case 4). Assuming the integral for the BHET is set to 1, Equation 3 simplifies the equation to indicate the ratio of protons.

$$n_{BHET,rxn} = \left(\frac{I_{BHET}}{N_{BHET}}\right) \cdot \left(\frac{N_{DPE}}{I_{DPE}}\right) \cdot n_{DPE} \qquad \text{Equation 2}$$

$$n_{BHET,rxn} = 2 \cdot n_{DPE} \cdot N_{DPE} \qquad \text{Equation 3}$$

To determine theconversion Equation 4, was used. Since only a fraction of the reaction mixture (0.1 mL) was placed into the NMR tube, this value had to be scaled to the volume of the reaction mixture (2 mL). Therefore, the ratio of $n_{\mathrm{BHET,rxn}}$ to $n_{\mathrm{BHET,expected}}$ was correcting for the volume of reaction in the NMR tube ($V_{\mathrm{rxn\ aliquot}}$) to the total reaction volume ($V_{\mathrm{rxn\ total}}$).

$$Conversion = \left(\frac{n_{BHET,rxn}}{n_{BHET,theo}}\right) \cdot \left(\frac{V_{rxn\ total}}{V_{rxn\ aliquot}}\right) \cdot 100 \qquad \text{Equation 4}$$

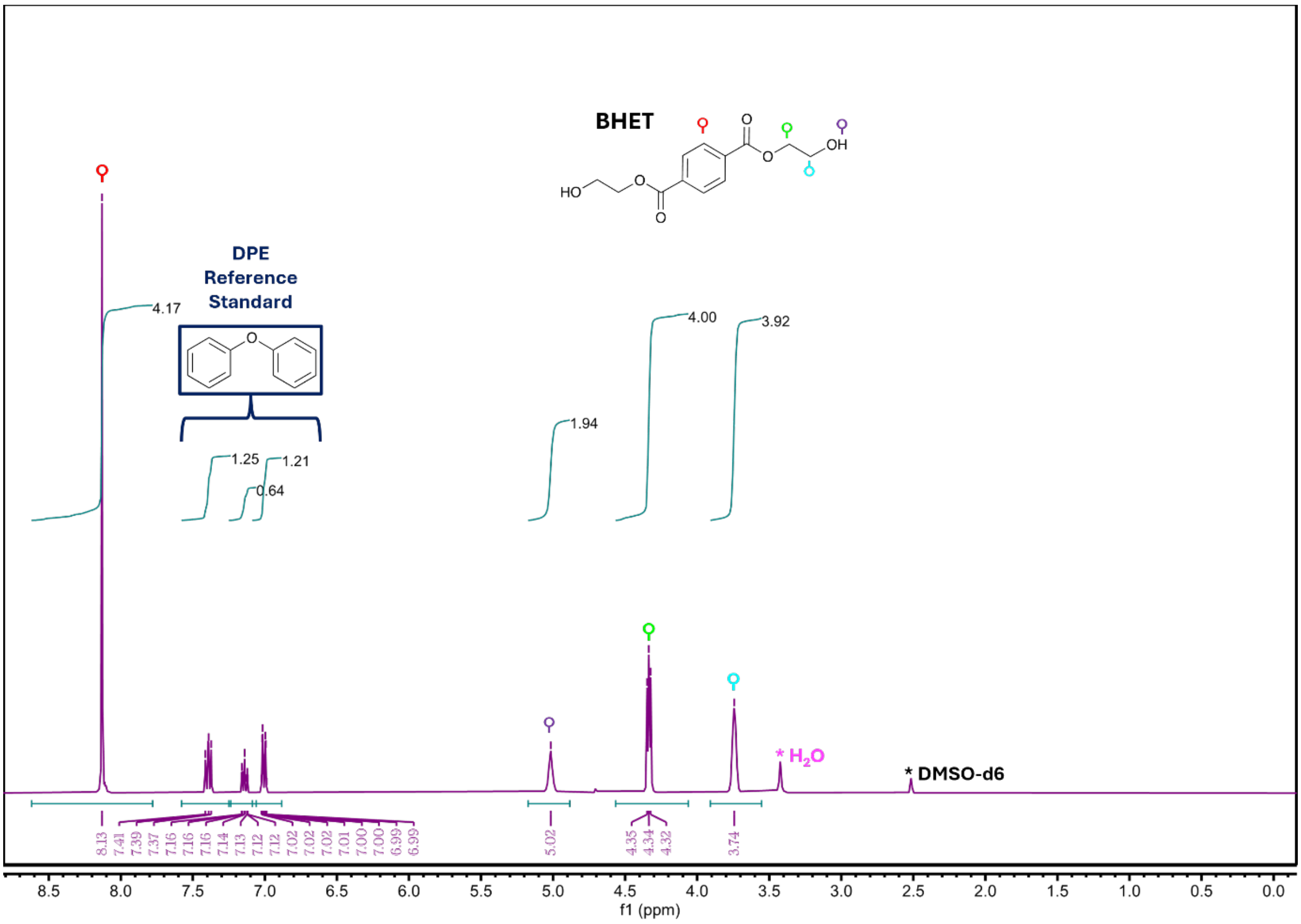

Figure S5. Representative NMR of frunk-s after glycolysis for 2 hours at 180 ºC, 5 eq. ethylene glycol, and 0.1 eq. DBU. The BHET yield was determined by using the integral of the DPE standard at 7.08 ppm and its known concentration and referencing back the integral at 8.13 ppm for the BHET, both normalized by the representative number of protons.

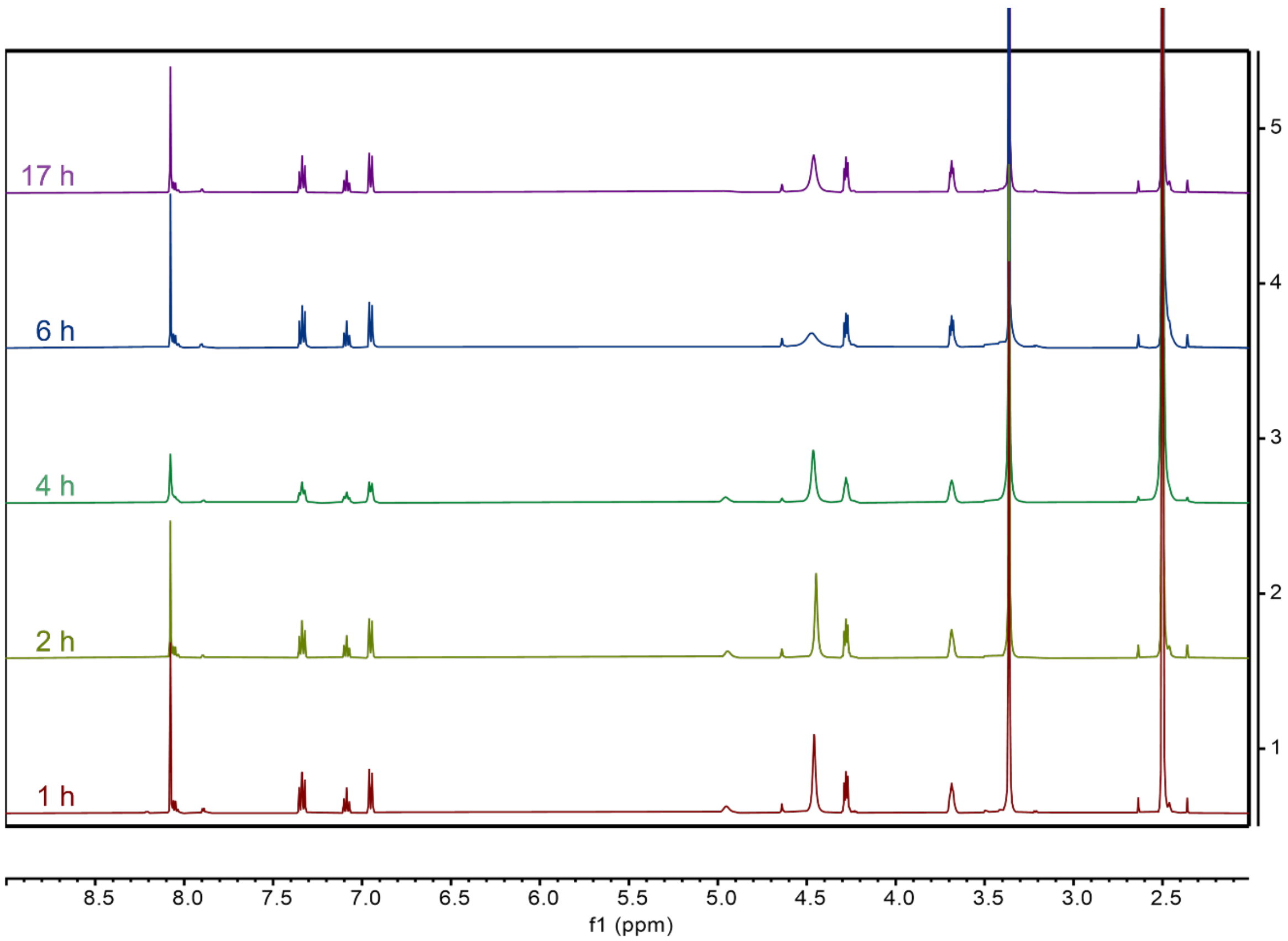

Figure S6. Stacked NMR plots for shredded frunk glycolysis at 5 time points, for the reaction with 5 eq. ethylene glycol and 0.1 eq. DBU catalyst at 180 ºC.

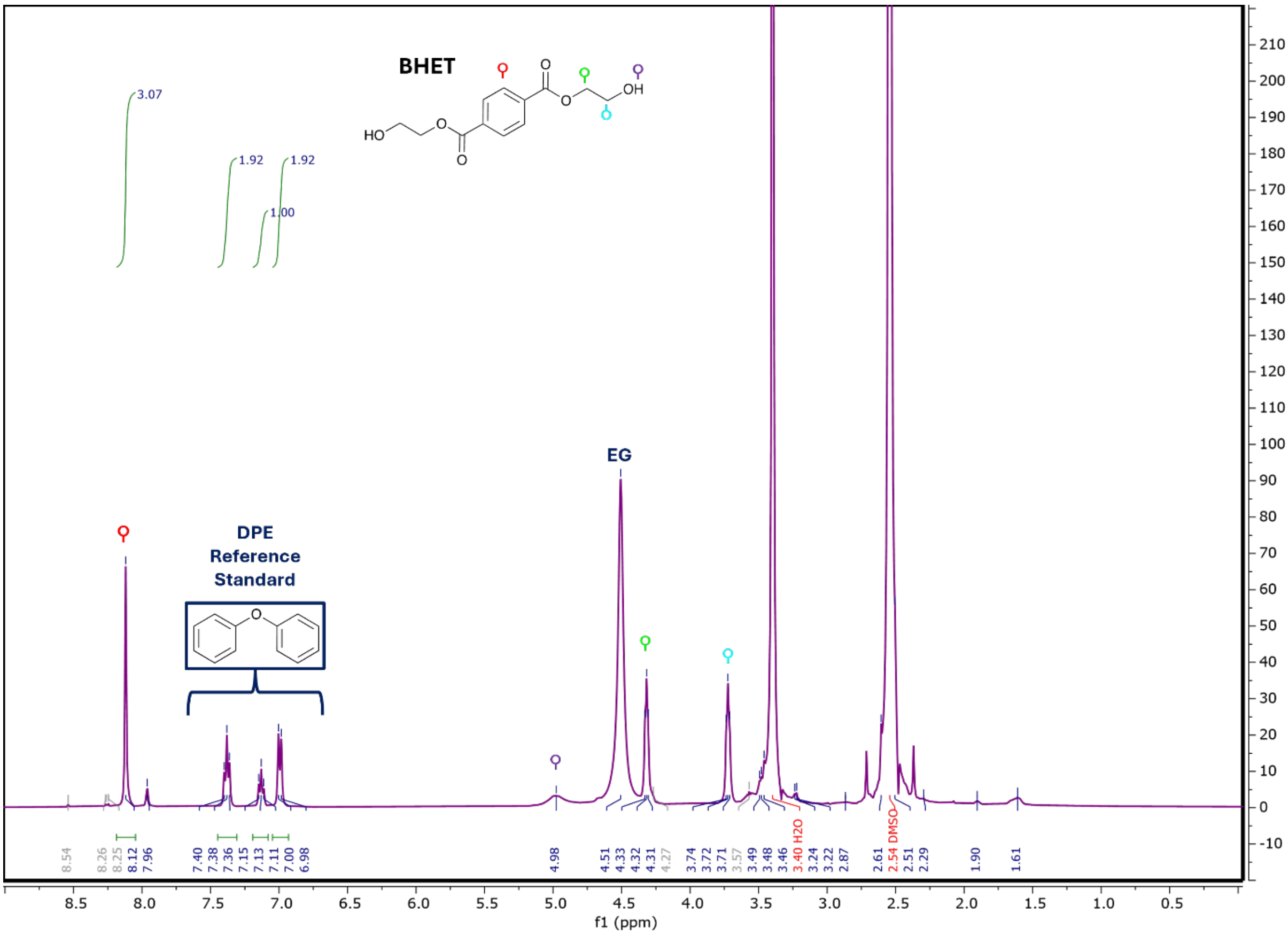

Figure S7. qNMR for a 10 L batch reaction with 1 kg PET, 10 eq EG, 10 mol% DBU loading.

2.1 Reaction optimization

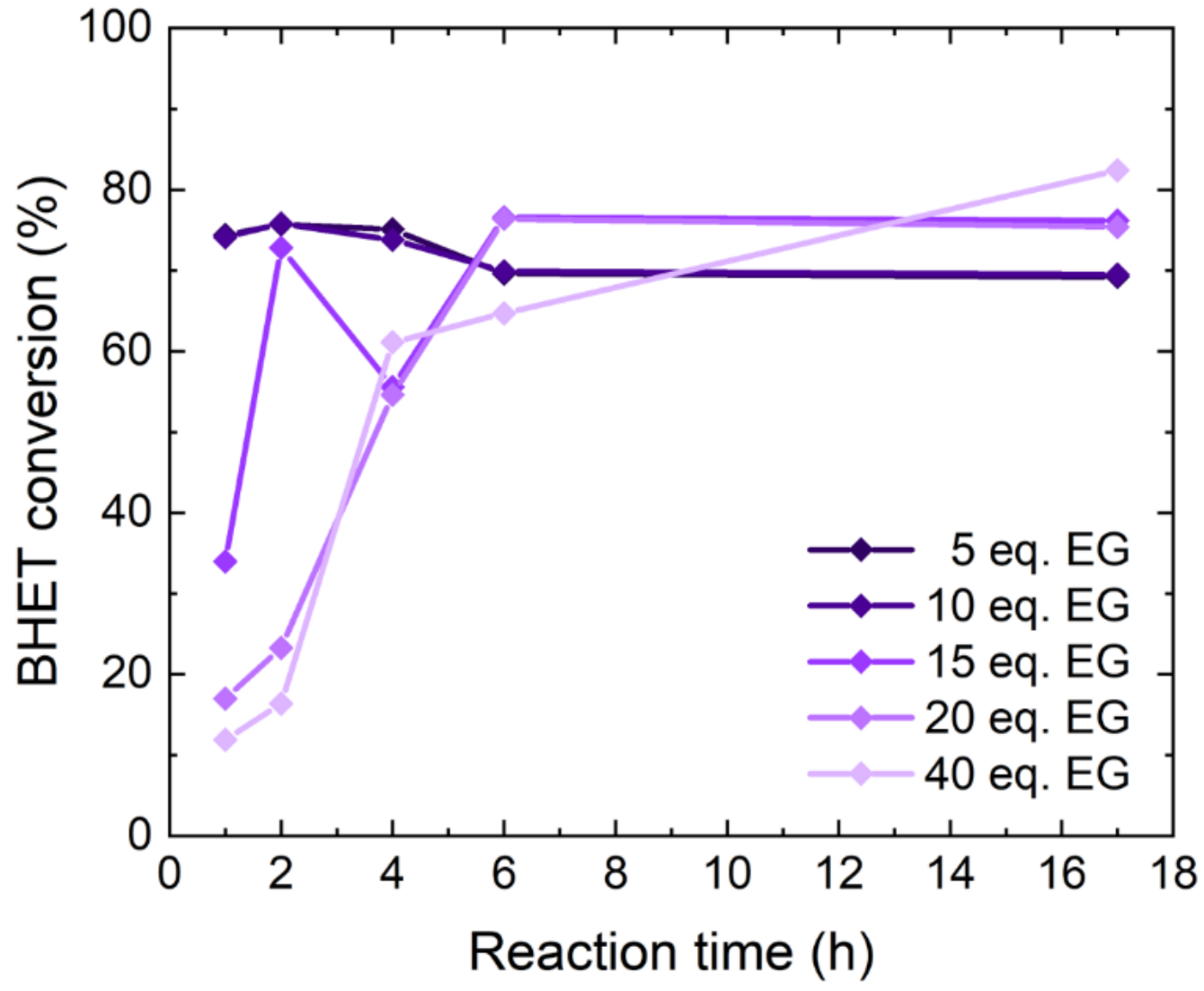

Figure S8. BHET conversion with time for varying ethylene glycol concentration for shredded frunk, at 0.1 equivalents of BHET. Concentrations range from 5 to 40 eq., with line color lightening with increased EG concentration.

2.2 Detailed scaled-up glycolysis procedures

A scaled-up reaction at 30 g of frunk PET (100x scale up, run twice, once with frunk-s and once with frunk-p) was performed in a round bottom flask (500 mL) at 180 ºC in an oil bath for 2 h while stirring at 500 rpm with a stirrer bar. To 30 g frunk was added 87.3 mL EG (10 eq.) and 2.33 mL DBU (0.1 eq.). As for the small samples, but more pronounced for the fluffy frunk flakes, the high volume of sample collapsed to form a black liquid. After stirring for 2 h, the mixture was taken off the oil bath for 20 minutes, before 300 mL of deionized water was added, in order to collect the BHET for further work up, following the work of Jehanno, et al. and Wood, et al [2,3]. As soon as water was added, the black solution turned gray as a result of the precipitation of white BHET mixed with carbon black. The mixture was then filtered with a Büchner funnel and the precipitate washed with 300 mL of 80 °C hot deionized water. The liquid phase was dried using a rotary evaporator at 50 °C and dried in a vacuum oven at 90 °C overnight to give a white crystalline product. The filter cake was dried separately and analysed by NMR.

Recrystallizations were performed on each sample as follows. 200 mL of ethylene glycol were added to a round bottom flask containing 30 g of BHET. The mixture was heated to 120 ºC and stirred for 30 minutes. The previous turbulent solution turned into an orange-gray transparent solution and allowed to cool down to 90 ºC before 80 ºC deionized water was added. Upon addition of the water, the BHET crashed out of the solution forming a white, flaky precipitate. The solution was then filtered with a Buechner funnel that was blocked from the precipitated white BHET. 100 mL of hot water were then added to wash the precipitate, but the filter cake could not be dried sufficiently.

For recrystallization, 300 mL of ethylene glycol were added to 60 g of BHET and heated to 120 ºC and stirred for 30 mins. The previous turbid solution turned transparent and was allowed to cool down to 90 ºC. Upon cooling, the transparent solution turned turbulent following the crystallisation of BHET. Hot water of 80 ºC was added and the solution allowed to cool down to room temperature and left to rest overnight. The next day, the solution was filtered, the filter cake separated and the filtrate cooled in the fridge for overnight to force further crystallisation of BHET from the solution. The respective filter cakes were then dried in a vacuum oven.

Since the water-workup lead to the loss of yield due to coprecipitation of BHET with carbon black in the filter cake, an approach to use DMSO instead of water was made. The intention was to dissolve the BHET only and not the non-reacted PET or carbon black. Instead of adding water after the reaction has been stopped, the reaction mixture was allowed to cool down before DMSO was added. A black liquid was obtained, after the BHET was dissolved. This mixture was then filtered through a Büchner funnel. Initially, carbon black went through the filter, before it accumulated and blocked the filter. The filter cake then served as an additional filter and allowed to collect an orange liquid without carbon black. However, BHET was also held back in the filter cake and crystallized separate from the filtrate. NMR was performed at each step to check for impurities and loss of BHET (Figure S11 and S12). Since the filtration did not work sufficiently, the process using water-workup was chosen as preferred workup method in further upscaling.

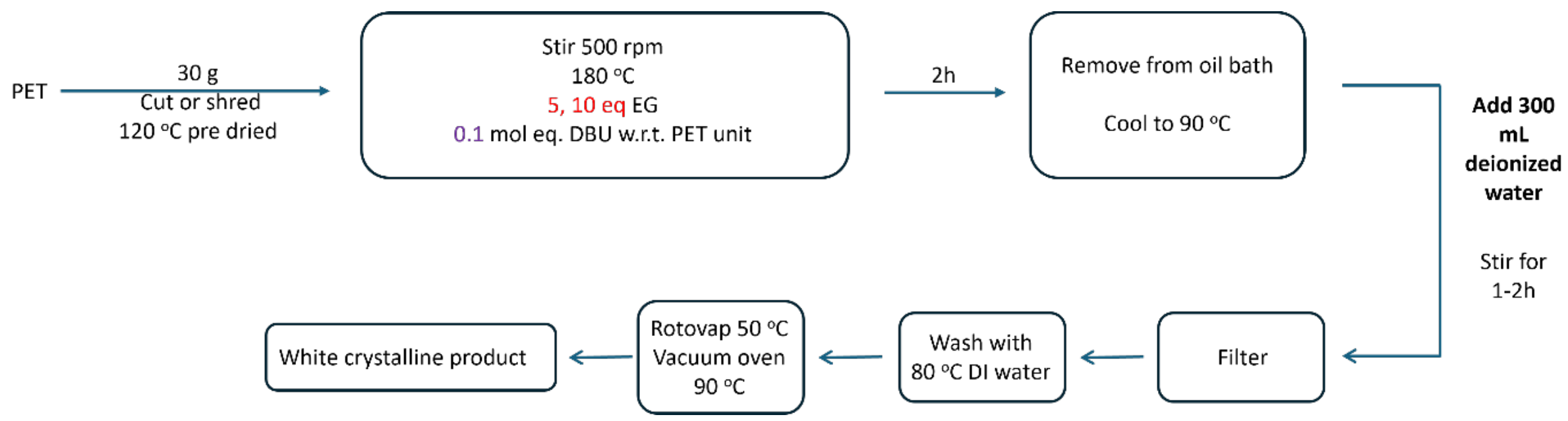

Figure S9. Schematic of reaction steps for 30 g scale reactions.

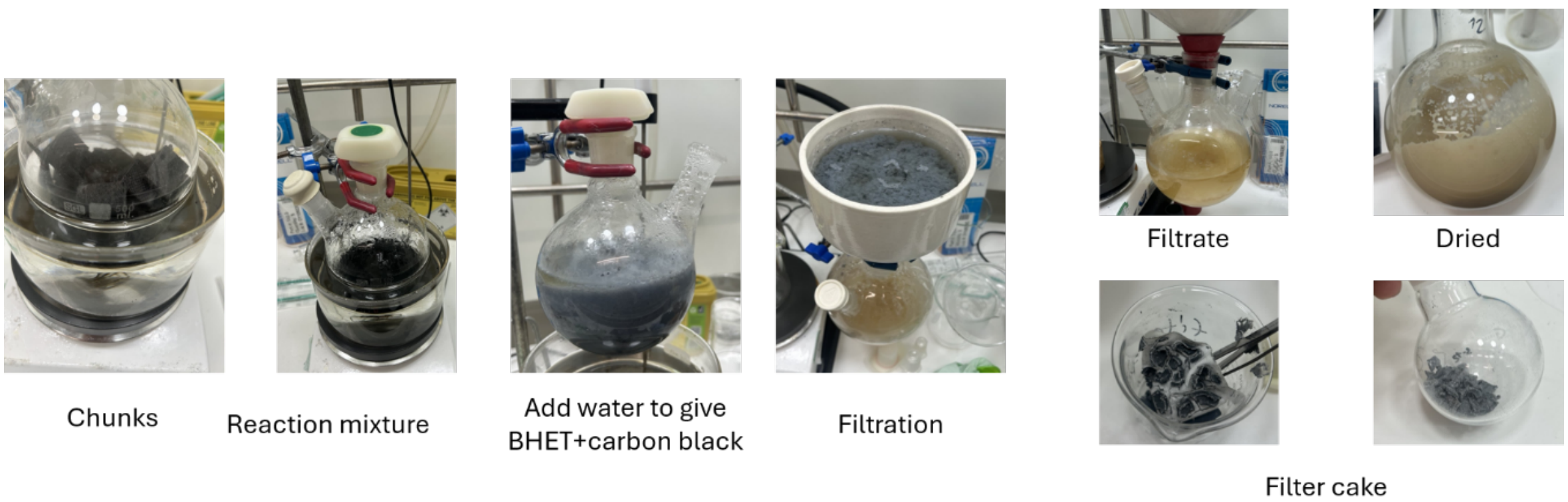

Figure S10. Workflow of the frunk-p 30 g reactions.

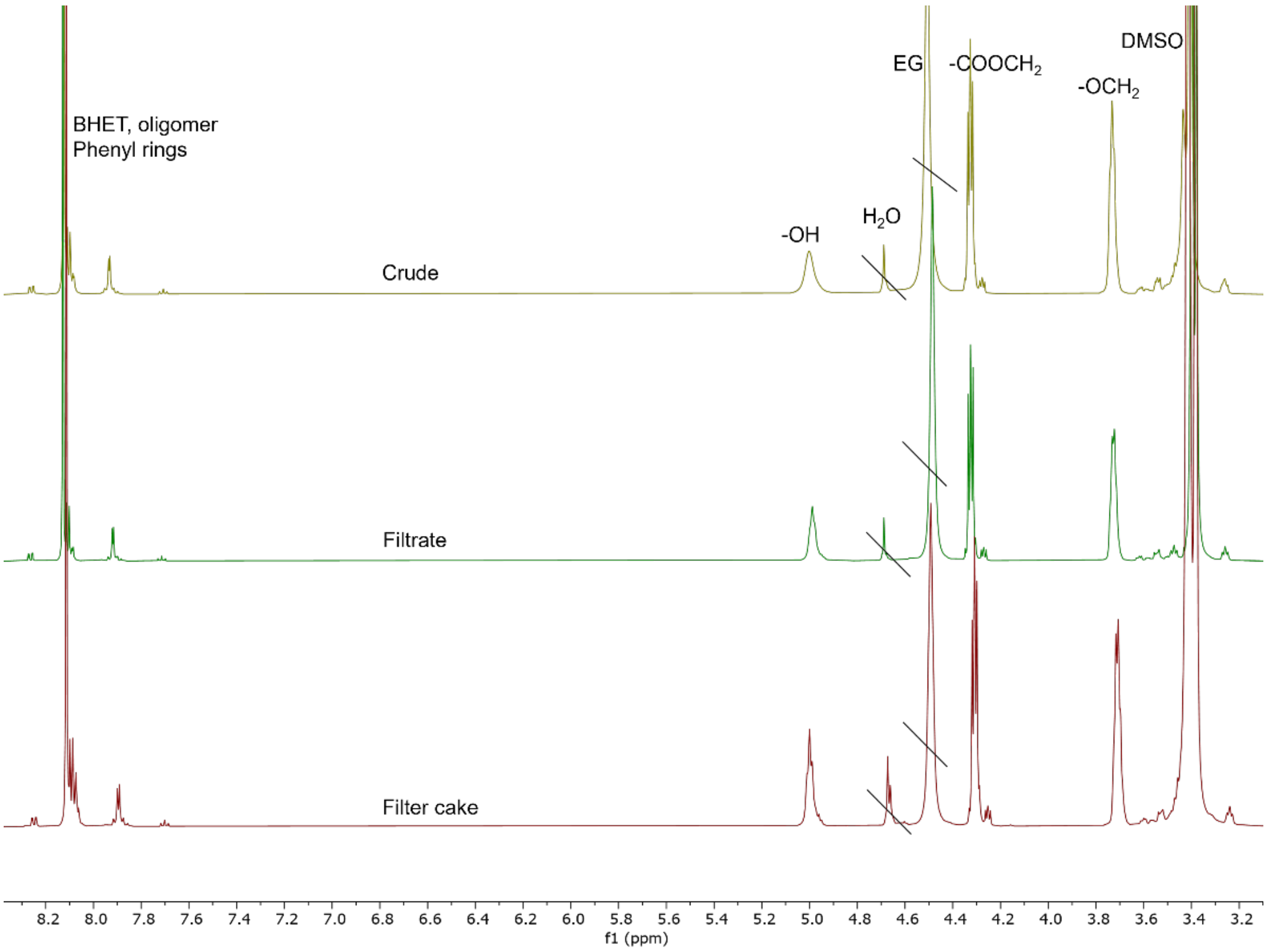

Figure S11. ^{1}HNMR of the 30 g reaction crude, after filtration, and of the filter cake containing carbon black. These show both that BHET is lost to the filter cake and that no organic additives were detected.

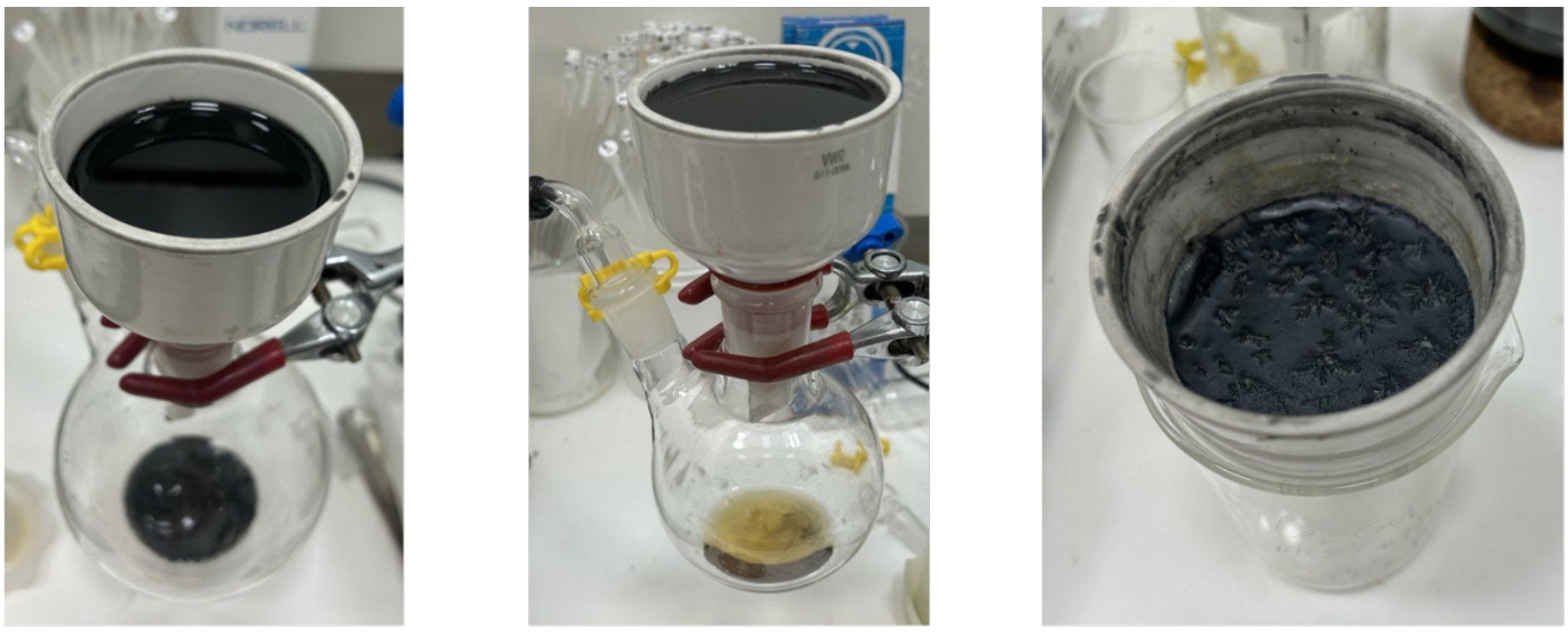

Figure S12: Filtration of a sample that was worked-up with DMSO instead of water. As depicted, the filtration was not successful until enough filter cake was formed to remove the carbon black from the reaction mixture. BHET crystallized upon drying of the filter cake.

To move to larger scales, a 10 L Syrris Orb Pilot batch reactor was used to run the reaction at the scale of a 1 kg of frunk liner, to which 3.3 kg (10 eq.) of EG was added along with 0.8 kg (0.1 eq.) of DBU. The reactor was fitted with a PTFE anchor stirrer shaft driven by a Heidolph

Hei-TORQUE 400 overhead stirrer and a condenser (deionized water and EG chiller), and filled with N_2. The external heating (Huber Unistat) was set to 180 ºC while degassing, which was reached after 1 h 40 min. Once the reaction temperature reached 170 ºC, the reaction was allowed to stir for 2 h at 200 rpm (180 ºC was reached quickly after starting). After 2 h, the reaction was allowed to cool to 90 ºC, a sample was removed from the reaction mixture for quantitative NMR analysis, and deionized water (4 kg, 3 eq. relative to theoretical BHET) was subsequently added. The water mixture was heated back up to 90 ºC and hot filtered over cotton wool and filter paper. The supernatant was allowed to cool to room temperature. The BHET that precipitated from the solution was vacuum filtered and washed twice with deionized water, and then analyzed by NMR spectroscopy and HPLC to determine the purity. 626 g of BHET was recovered from the reaction.

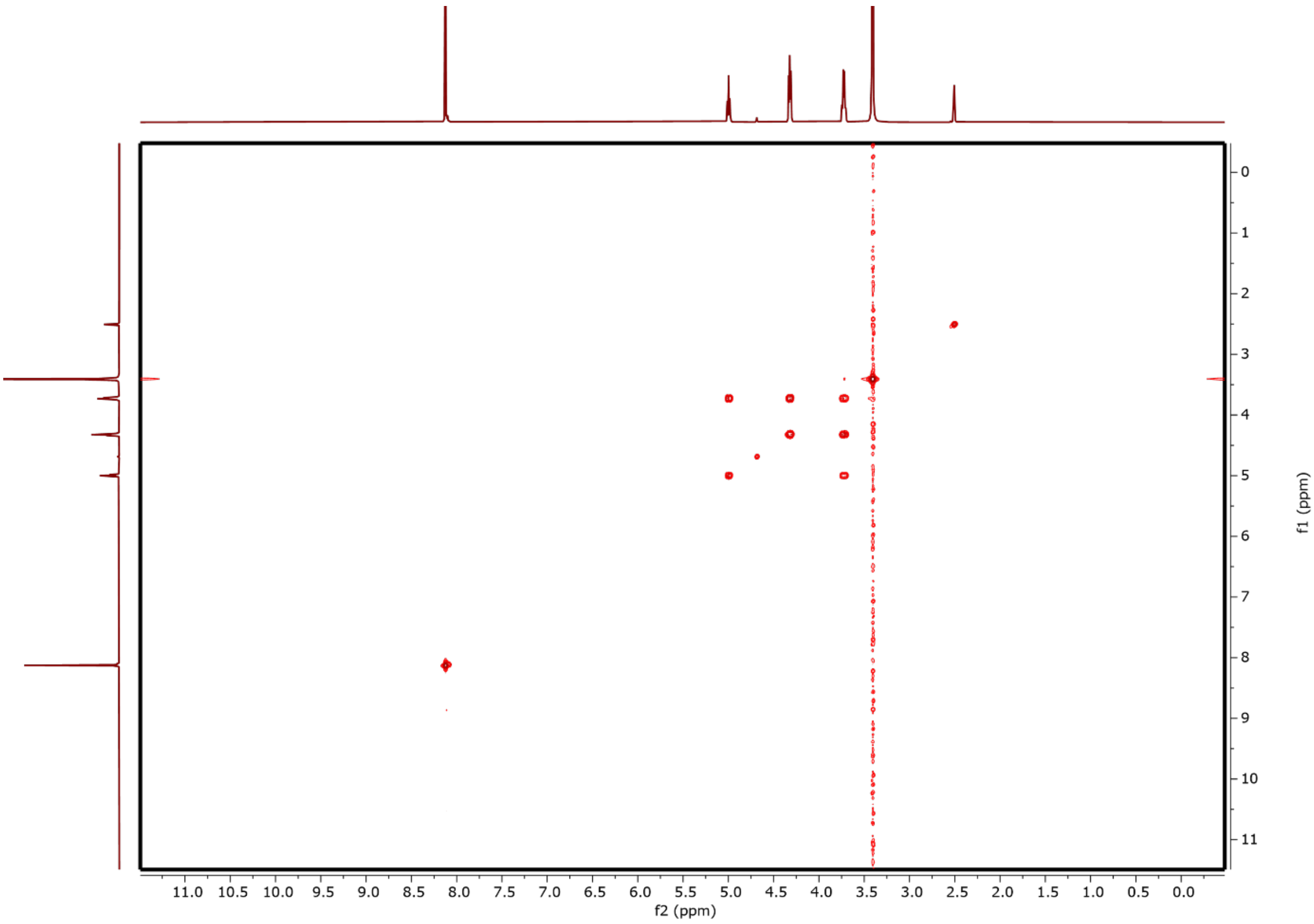

Figure S13. 2D COSY NMR of the purified product from the 1 kg reaction. All detected peaks correspond to either BHET, DMSO, or H_2O in the DMSO-d_6. Peak correlation is between neighbouring protons on BHET, with no detected contaminants.

Note that at this point the following impurities might be present in the BHET, but their presence could reliably be excluded: (i) ethylene glycol (EG) which would suppress crystallization of BHET but should be removed by the cold water washing step. Whilst EG would appear in the NMR, at a chemical shift of BHET and therefore with the potential to interfere with the determination of conversion, the match of integral intensities of the main signals of BHET suggest a high purity of the deconstruction product. (ii) Terephthalic acid or MHET would give multiple signals in the aromatic region of BHET and alter the integral intensity of the ethylene signals and the aromatic signals of BHET. However, from ^{1}HNMR of the crude reaction (Figure

S5), the integral relationships between alcohol and aromatic signals correspond to di-substitution of the terephthalate ring. (iii) Carbon black would be apparent in the color of BHET, but since a white powder was formed, a sufficient removal of carbon black was assumed. (iv) Additives, degradation products and catalyst would show up in the NMR, but were not present in significant concentrations.

To confirm the purity of BHET, high-performance liquid chromatography (HPLC) was performed on the purified samples from the frunk glycolysis and a commercially available BHET obtained from Sigma Aldrich. Samples were dissolved in DMSO to obtain a concentration of 5 mg/ml and injected into an Agilent 1260 Infinity II setup with an autosampler, a quaternary pump with a flow rate of 1 ml/min at 22 ºC was used. An C18 column of HyperClone-Phenomenex BDSC18 (H18-052044) was used for separation and the signal of a diode assay detector (DAD) at 254 nm used to detect the elution of the BHET. Water with 0.1 % formic acid were used as solvent A and MeOH was used as phase B. The following composition of the mobile phase was used: a steady composition of 90/10 (A/B), followed by a linear gradient over 10 mins to a composition of 10/90 of the mobile phase, then for 1 min a gradient back to a composition of 90/10 and finished with a steady composition of 90/10 for 15 mins.

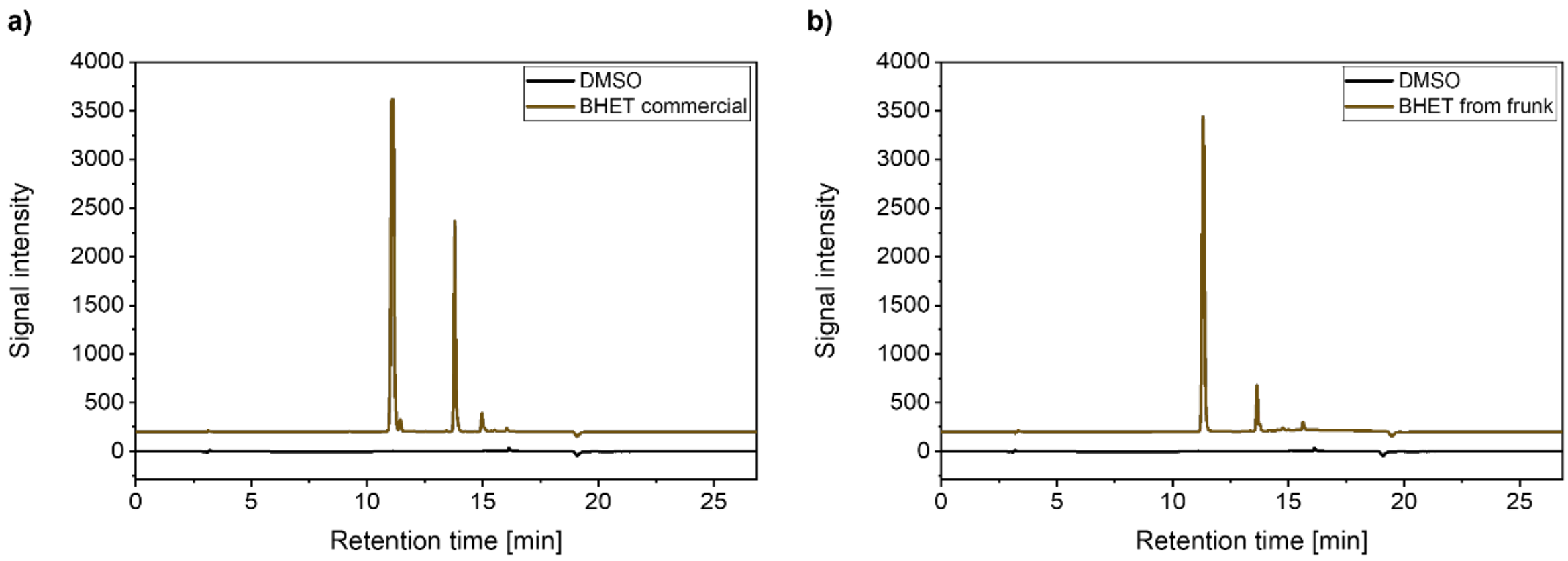

Figure S14: HPLC with a gradient of MeOH and water of: A) commercially available BHET and B) BHET from the depolymerized frunk.

As depicted in Figure S14, a similar chromatogram for the commercially available and the BHET from glycolysis was obtained. The similar shape of the chromatograms suggests that both samples have the same chemical compositions and the workup of the BHET successful. No additives or significant traces of impurities were found. Whilst three distinct signals were observed in the chromatograms (most likely BHET, MHET, TPA) this was either a result of hydrolysis of BHET to MHET or TPA within the column and the separation process, or products from oligomerisation. Note, that BHET seems to consist mainly of one component verifying the results from ^{1}HNMR spectroscopy.

2.3 Quantifying carbon black additives

To identify the amount of non-soluble additives and carbon black, three samples were depolymerized. 1.0 g of PET was deconstructed using the procedure described above. For that, the respective sample was mixed with 0.86 mL of a mixture of DBU in ethylene glycol (5 wt.%) and stirred for 2 h at 180 °C. After cooling down the reaction mixture, 10 mL of DMSO were added and the solution stirred for 2 h to enable a complete dissolution of BHET. Samples were transferred into an Eppendorf-tube and the reaction vessel rinsed with 2 mL of DMSO. 20 mL of isopropanol were added to the black solution and the mixture centrifuged with an Eppendorf Centrifuge5810, 4000 rpm for 20 minutes. The liquid phase (BHET, isopropanol, DMSO) was decanted off and the BHET allowed to crystallize overnight in a fridge at 2 °C. The solid phase was mixed with 25 mL of isopropanol, shaken and centrifuged again to make sure all DMSO was replaced by isopropanol. After discarding the liquid phase, the carbon black was dried in a vacuum oven at 50 °C overnight.

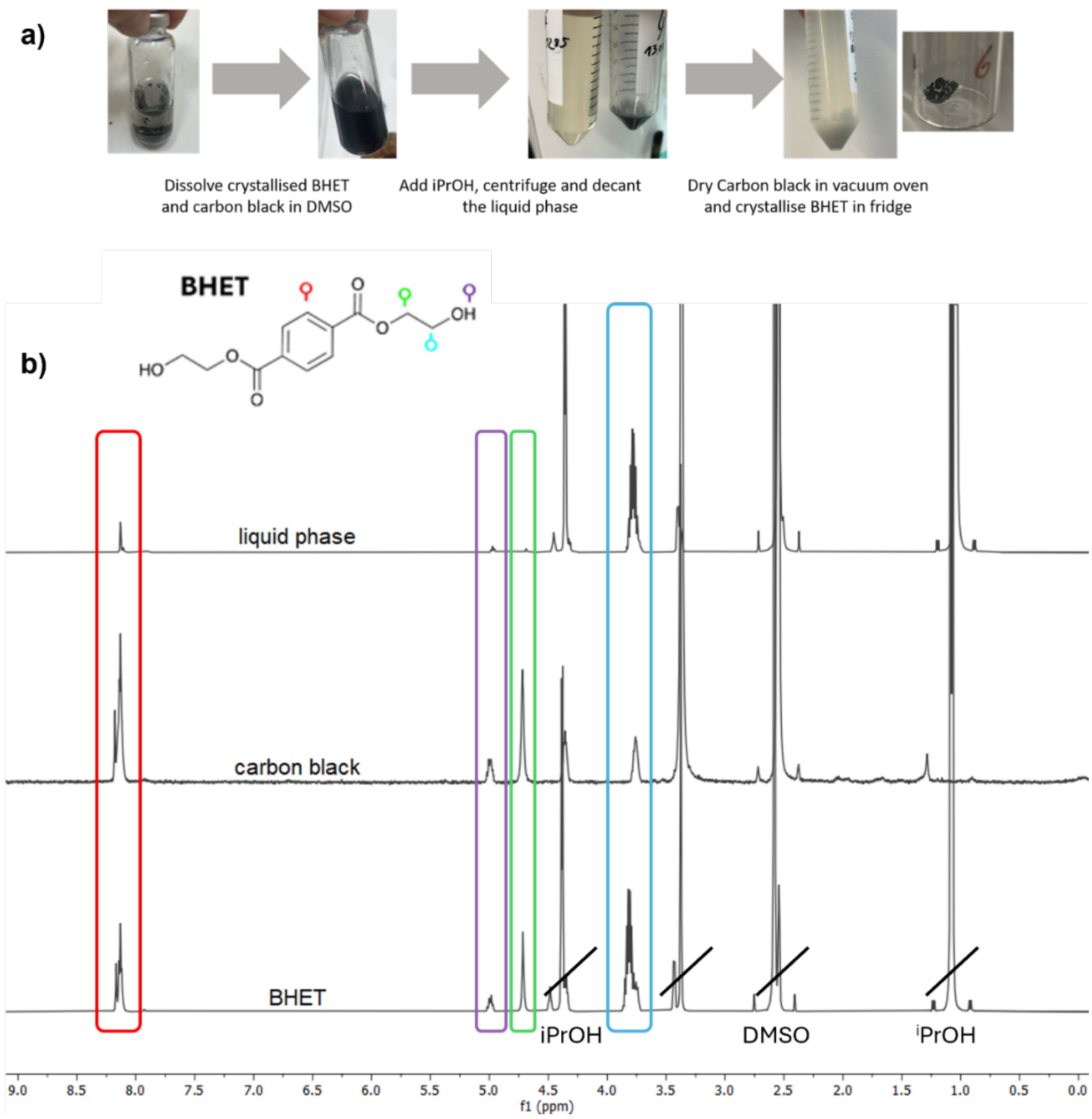

Figure S15: a) Process of carbon black separation using centrifugation: Dissolve reaction product, centrifuge off the carbon black and isolate BHET from the liquid phase. b) ^{1}HNMR of the samples obtained within the workup procedure focussing on carbon black: the carbon black fraction contained (likely) only traces of BHET, the crystallized from the liquid phase was mostly BHET and in the liquid phase, mostly BHET of a lower concentration was found.

Whilst the mass of the carbon black deviated between 5 and 15 mg, due to the nature of the additive, not all the carbon black could be recovered from the reaction vessel and the stir bar. Therefore, the amount of carbon black was likely underestimated, but should be in the range of 20 mg. The amount of carbon black aligns with the estimated values of 2 % obtained from TGA. To determine, whether more unknown organic additives were removed in this separation process, ^{1}HNMR spectroscopy of the respective fractions was performed.

As depicted in Figure S15b, all the samples consisted mainly of BHET. Considering that in section 2.1 and Figure S6, similar results were found, this reiterates that the frunk-PET consisted of PET and carbon black only. Organic additives of relevant concentration would have been found in the NMR spectrum, while inorganic or non-soluble additives would have been captured within the carbon black fraction.

3 Thermal properties

Thermogravimetric analysis (TGA)

The TGA was performed from 40 ºC to 600 or 1000 ºC at 10 ºC min^{-1} on a TA Instruments Discovery SDT 650 in a nitrogen atmosphere on either virgin PET pellets or shredded frunk. The residual material after PET had been removed from the system was determined to be carbon black, at an average of 2 wt% content relative to the virgin PET residue.

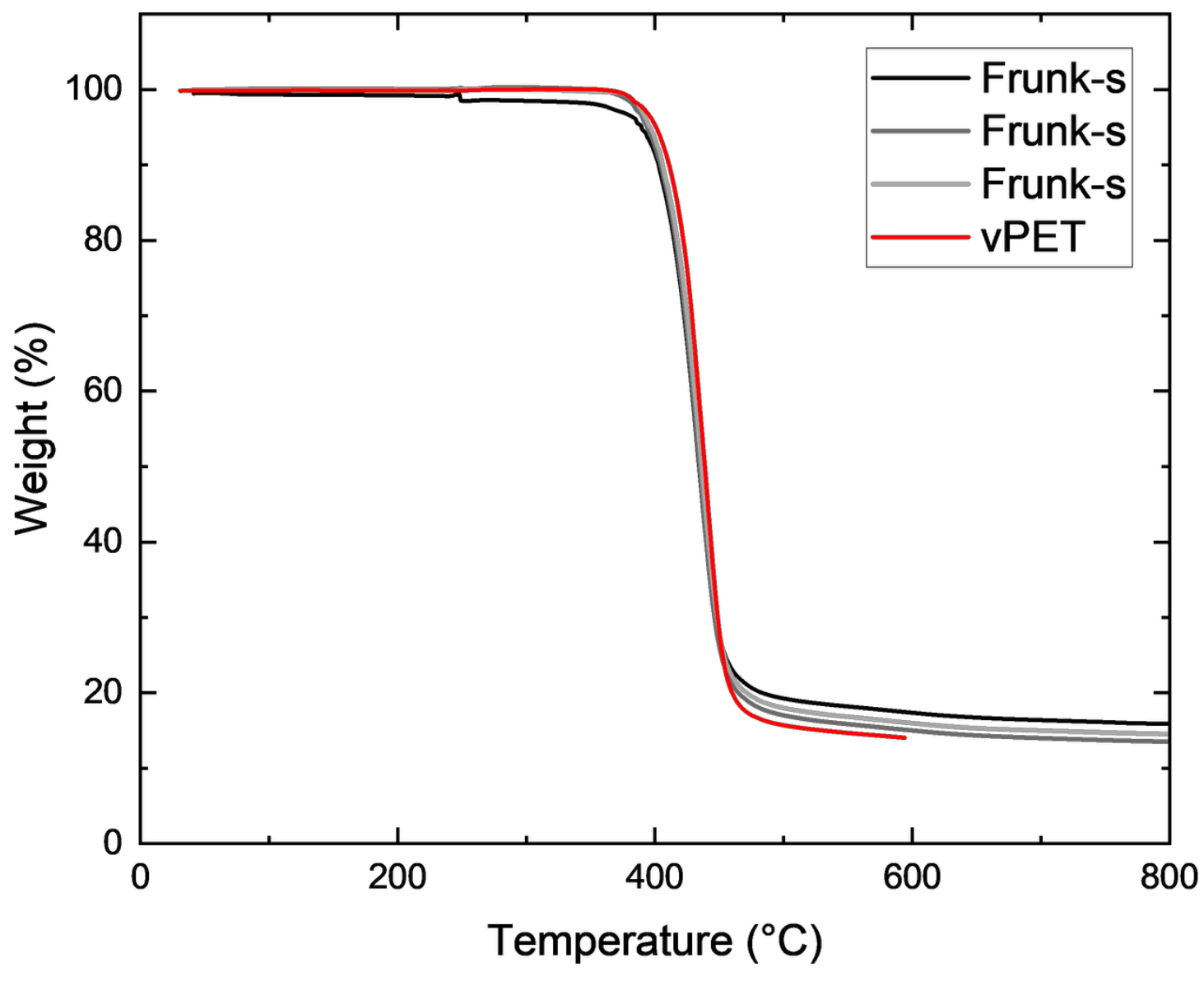

Figure S16. Thermogravimetric analysis (TGA) in N_2 of shredded frunk material and virgin PET. On average, the difference residual material between the frunk and the vPET after the PET has fully burned off makes up 2.2 ± 0.5% of the total mass, indicating that carbon black makes up 2.2% of the frunk material.

4 Effect of sample density on PET conversion

Table S2. Depolymerization of various geometries of PET. Chopped, shredded, and shredded/granulated frunk all depolymerized to 80% conversion in five minutes. Extruded frunk reached 33% conversion in that time, while vPET pellets only reached 17% conversion and bottle flakes about 60%.

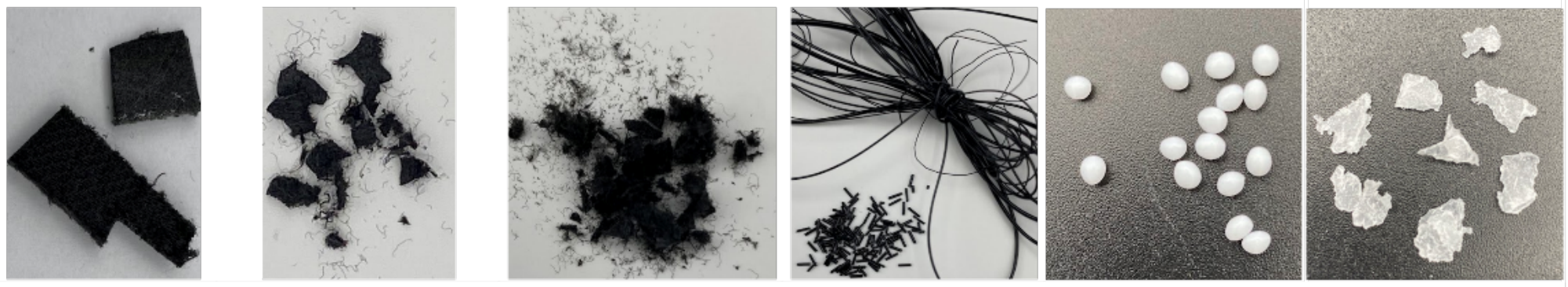

Material	Chunks	Shredded	Granulated	Extruded	Pellets	Flakes
Reaction time (min)	5	5	5	5	5	5
BHET conversion (%)	79.2	84.4	81.2	32.9	16.6	62.3

References

1 Jang JY, Sadeghi K, Seo J. Chain-Extending Modification for Value-Added Recycled PET: A Review. *Polym Rev* 2022; **62**: 860–889.

2 Jehanno C, Flores I, Dove AP, Müller AJ, Ruipérez F, Sardon H. Organocatalysed depolymerisation of PET in a fully sustainable cycle using thermally stable protic ionic salt. *Green Chem* 2018; **20**: 1205–1212.

3 Wood J, Grause G, Sutton J, Hmayed AA, Dove A. A Scalable Process for the Depolymerisation of Polyethylene Terephthalate (PET). *ISCRE28* 2024.https://www.iscre28.org/abstracts/abstract_96_35_1.pdf.
